# The EGPRN Research Strategy for general practice in Europe

**DOI:** 10.1080/13814788.2022.2080815

**Published:** 2022-06-06

**Authors:** Claire Collins, on behalf of the EGPRN Strategy authorship group

**Affiliations:** a Irish College of General Practitioners, Dublin, Ireland; b European General Practice Research Network, Maastricht, The Netherlands

**Keywords:** Research strategy, general practice/family medicine, research capacity building, knowledge transfer and exchange, stakeholder involvement

## Abstract

The European General Practice Research Network (EGPRN) has recently published an updated research strategy with the overall aim being to promote relevant research of the highest quality within general practice/family medicine (GP/FM). The Research Strategy indicates a global direction and serves as a basis for more detailed plans in individual countries that will take into account the characteristics of a country, its specific needs and the level of current research capacity. This paper aims to provide a summary of the EGPRN Research Strategy.

The Research Strategy suggests that it is necessary to consider what the knowledge deficits are and to set research priorities. Research capacity building (RCB) is required at all levels. Research in GP/FM will also have to reflect the changes in the profession. An innovative and sustainable-oriented approach to conducting research is needed. Use of existing toolkits and engagement with patient platforms and representative groups are necessary to ensure meaningful user involvement. Knowledge transfer and exchange (KTE) is an important component to ensure a process of exchange between researchers and knowledge users.

Working to improve leadership, to support the creation of a research culture in GP/FM and to increase national and international networking are considered as fundamental to ensuring a portfolio of high-quality research and for improving the impact of GP/FM research. The recommendations in the Research Strategy are based on a review of the literature on general practice research from 2010 to 2019 and are set in the context of a theoretical framework.

Box 1. The four overarching goals of the Research Strategy.

- To identify priorities for general practice/family medicine research
- To support research capacity building
- To develop and promote high standards of research practice
- To foster the translation of evidence into practice

KEY MESSAGES

- Working to improve leadership, to support the creation of a research culture in GP/FM and to increase national and international networking are fundamental to ensuring a portfolio of high-quality research and improving the impact of GP/FM research.
- The EGPRN Research Strategy indicates a global direction for research in GP/FM and serves as a basis for more detailed strategic plans in individual countries.
- You should utilise the Research Strategy by selecting the appropriate recommendations relevant for your country's GP/FM context and stage of development.

## Introduction

In 2009, the first European General Practice Research Network (EGPRN) Research Agenda was published [1]. It was developed at the request of WONCA Europe, was related to the European Definition of General Practice/Family Medicine (GP/FM) [2], and was based on several key informant surveys and a comprehensive review of the scientific literature. The EGPRN has recently published an updated research agenda, now framed as a research strategy – EGPRN Research Strategy for General Practice in Europe 2021 (further referred to as ‘Research Strategy’) [3] [see supplementary file]. This paper aims to provide a summary of the EGPRN Research Strategy for General Practice in Europe.

The Research Strategy provides an overall plan with guidance to achieve four specific goals, namely, to set research priorities, build research capacity, promote high standards of research practice and foster the translation of evidence into practice (Box 1).

Box 2. Measuring the impact of a research strategy.

The impact of a research strategy can be measured in terms of:

- capacity building
- scientific productivity (dissemination of research results)
- establishment of networks and collaborations
- involvement of stakeholders and policymakers
- involvement of the population
- improving the health of the citizens

The vision for the Research Strategy is to promote relevant research of the highest quality within GP/FM where an evidence-based culture informs efforts to improve the health of citizens and to develop services. The mission statement of the strategy presents how we can achieve this by contributing to the strategic development and growth of research and innovation across GP/FM in Europe. A key basis for the strategy and its recommendations is that a robust research basis is a necessity for GP/FM [4,5]; and that high-quality research in general practice is an important component in improving patient outcomes [6].

## Rationale for the Research Strategy

The rationale for the Research Strategy is outlined clearly (Chapter 1 of the Research Strategy [3]). According to the World Health Organisation (WHO) Declaration of Astana [7], primary care is crucial for the health system, and enhancing the capacity and infrastructure of primary care is a key task for governments worldwide. Information is increasing rapidly due to advances in technology, which imposes new challenges on primary health care [8]. Real World Evidence (RWE) and Real World Data (RWD) are playing an increasing role [9]. Information systems have to support the collection of data to monitor the performance of the health system according to all of the criteria for good health care [7].

In the past ten years, many changes have challenged the validity of the WHO’s definition of health in the 21st century [10]. A new way of viewing human health has emerged [11] and this requires a more dynamic formulation in terms of organisation and delivery of services [12]. New models of health care delivery have been developed in primary health care, including integrated primary care [13]. These primary care models need strong linkages with public health, community services, and other sectors that address the social determinants of health [14]. GP/FM interventions need to sufficiently incorporate this perspective while ensuring effectiveness, safety, and patient-centeredness. Research in GP/FM will also have to reflect these changes in the profession.

New practice and research models, such as the Care and Learn Model [8], are proposed to help identify research gaps and improvement opportunities, evaluate existing programs, inform priority setting, and develop effective responses to the evolving needs of a rapidly changing healthcare landscape [8].

The EGPRN Executive Board considered that ten years on from its Research Agenda [1], it was time to review developments and assess the current landscape, moving to a Research Strategy to advance from presenting a series of problems and related actions to providing an overall strategic plan to achieve specific goals (Chapter 2 of the Research Strategy [3]), which can be adapted to the needs in each country.

## The foundations underpinning the Research Strategy recommendations

**Figure 1.**
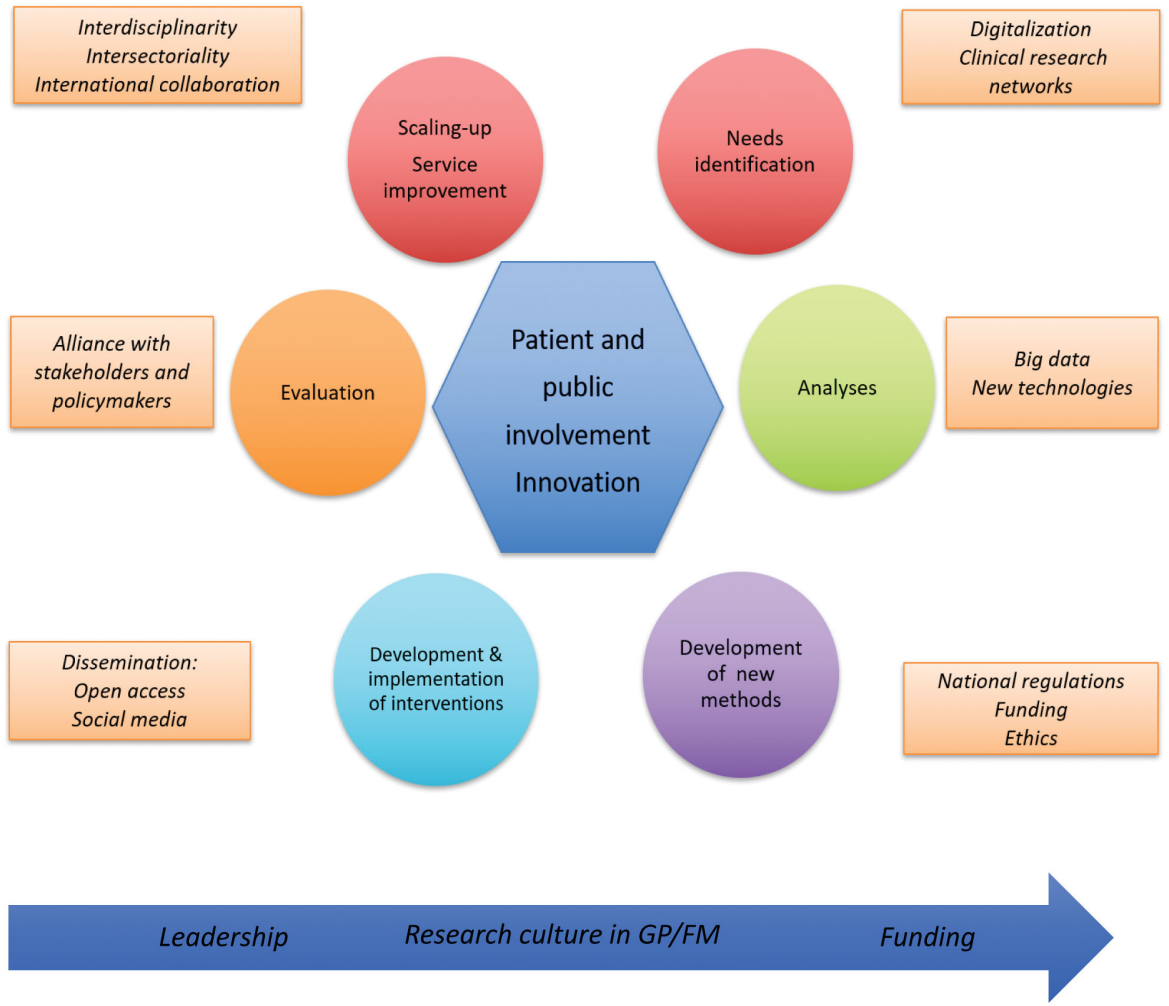
Theoretical framework for the Research Strategy recommendations. Developed based on previous work [16,17].

The recommendations in the Research Strategy are based on achieving four goals (Box 1) and were developed based on:

- a bibliographic search from 2010 to 2019 using the keywords considered most relevant to the recommendations of the first EGPRN research agenda (Chapter 3 of the Research Strategy [3]);
- a review of the high impact papers in GP/FM in terms of the most cited articles over 2010–2019 (Chapter 4 of the Research Strategy [3]);
- the review of a sample of EGPRN conference abstracts as an indication of research taking place and being disseminated in the GP/FM community (Chapter 4 of the Research Strategy [3]); and
- a proposed theoretical framework through which to understand the relationships between the recommendations proposed (Figure 1) (Chapter 5 of the Research Strategy [3]).

The detailed methodological approach for each of the above is outlined in the Research Strategy [3], included as a supplementary file.

### The literature search and EGPRN abstract review

A bibliographic search was conducted based on the final recommendations in the previous research agenda [1], using the keywords considered most relevant to these recommendations (Table 1).

**Table 1.** Bibliographic search summary.^f^

	Broad topic areas	Total articles	Clinical trial/RCT	Systematic review/ meta analysis	Observational study/review
Instruments/outcome measures^a^	Primary care management Patient-centred care Specific problem-solving competency Comprehensive and holistic approach Community orientation	1,008,543	132,606	30,539	95,330
Education and training^b^	Patient education Training methodology Sustainability	45,481	7,623	2,492	6,235
Preferences and perceptions^c^	Patient preferences Doctor perceptions Communication/Communication skills Patient involvement Public involvement Shared decision making Self-management Decision support Stakeholder engagement	36,248	5,706	1,993	5,276
Evaluating effectiveness/efficiency^d^	Patient-centred approach Comprehensive approach Biopsychosocial care Community orientated healthcare Integrated primary care	21,847	6,614	2,037	4,214
Databases/electronic records^e^	Research based on registries	16,443	1,082	4,776	4,546

^a^Developing and validating instruments and outcome measures for each competency or domain (or components thereof), taking into account their complexity and interactions.

^b^Developing methods of education and training for components of the different GP/FM competencies and evaluating their effectiveness, including the impact on health care and health outcomes, in the short and long term.

^c^Studying patients’ and doctors’ perceptions, perspectives and preferences regarding specific components or aspects of each research domain.

^d^Evaluating effectiveness and efficiency of a patient-centred approach, a comprehensive approach, a biopsychosocial care model, and community orientated healthcare (as compared to a biomedical and specialist approach), including different models or management strategies.

^e^Developing primary care databases as a basic infrastructure for both health care and research, including studying and improving the utility and validity of data from electronic patient records in GP/FM.

^f^The search was conducted in PubMed for the period of 1 January 2010 to 31 December 2019. The full search terms are outlined in the Research Strategy document (see supplementary file) [3]. All searches were restricted to publications with an English language abstract available. Articles from the United States, Asia, Australia, New Zealand, Russia, Africa, and South America were excluded. The following terms were applied to all searches: Primary health care OR Family Practice OR General Practice OR Family Physician.

The results show the highest number of publications related to instruments and outcome measures for each competency or domain of general practice. It was noted that a high number of publications included the perspectives of doctors and/or patients; and to analysing efficacy. Shared decision making, stakeholder engagement and patients’ preferences were least often included. It was clear from the search that general practitioners/family physicians are incorporating big data, *via* electronic medical databases, into their research, although this has not yet extended to all countries. The number of clinical trials, systematic reviews and meta-analyses to generate evidence in GP/FM were noted to have increased somewhat.

In terms of the papers with the most impact in the field since the last strategy was published, the Web of Science was searched for the most cited articles using the terms Family Medicine or General Practice or Primary Health Care as the main subject, excluding editorials in the period 2010–2019. Among the top 10 papers, five were cross-sectional studies in the form of surveys, observed behaviour and use of registers, two were reviews of literature, one presented cost-effectiveness analyses and two papers described the effect of interventions. Thematically, most papers dealt with clinical topics, followed by papers studying the medical encounter and family medicine as a profession. Citations numbers of the most cited papers were under 200 over 10 years.

From the sample of EGPRN abstracts reviewed, descriptive and cross-sectional studies using both qualitative and quantitative techniques were the most common. Little evidence of clinical research in terms of interventions was noted, with few randomised controlled trials (RCTs) and even fewer studies regarding feasibility, efficiency or efficacy. User involvement in a broad way, apart from their participation as study subjects, was seldom reported. A relatively high proportion of research in the abstracts reviewed dealt with topics related to GP/FM as a discipline.

### A theoretical framework

The implementation gaps between evidence-informed interventions and their delivery include adapting and scaling effective interventions to bridge the transitions from evidence to policy, policy to implementation and implementation to system quality [15]. However, the relationships between research, policy and practice are complex and might not be possible to disentangle. Our theoretical framework is adapted from the wheel of knowledge developed by Stange [16] and recognises that the research in general practice is derived from practice experience [17]. This theoretical framework provides a model to inform and link the Research Strategy recommendations (Figure 1).

## Discussion

Chapter 6 of the Research Strategy [3] outlines the way forward in terms of recommendations for future action and suggests how the success of these might be measured.

The impact of health research is measured in terms of its translation into clinical practice, health policy and behavioural change. The impact of a research strategy can be measured in terms of capacity building, scientific productivity (dissemination of research results), establishment of networks and collaborations, involvement of stakeholders and policymakers, involvement of the population and eventually in improving the health of the citizens (Box 2). The EGPRN have devised outcome measures through, which they will measure the impact of their Research Strategy. Each country should ensure to specify outcome measures so that it is possible to track the success of actions implemented.

### Research capacity building

If we are to continue to improve the quantity, quality, impact and coordination of GP/FM research, a key concept noted is that of capacity [18]. Research capacity building (RCB) is critical at the individual, organisational and environmental levels [19]. Working to improve leadership, to support the creation of a research culture in GP/FM and to increase international collaboration and networking are fundamental in this regard.

### Knowledge deficits, research priorities and research networks

We must also establish our knowledge deficits and to set research priorities [20,21]. Ideally, these should be agreed, prioritised and coordinated at a national and international level [20–22]. GP/FM researchers need to establish and engage in both national and international networks to successfully deliver a portfolio of high-quality studies [18,23,24]. Collaborative funding proposals on a European level should be sought. However, responsible innovative approaches must be adapted to different settings and health systems, taking account of the specific context [25,26].

### *User involvement*, *knowledge transfer and exchange*

Meaningful user involvement [16] is yet to become the rule rather than the exception in GP/FM research. User involvement that reflects the diversity in our communities is required [16,27]. Participatory health research and specific approaches such as Participatory Learning and Action research (PLA) [28] can enhance patient engagement and support implementation and the explicit reporting of the procedures used are recommended [27]. Use of existing toolkits and engagement with national and international patient platforms and representative groups are necessary [29]. Also, knowledge transfer and exchange (KTE) is an important component to ensure a process of exchange between researchers and knowledge users in broader terms [30]. The generation of knowledge alone is insufficient to facilitate a more productive interface between researchers and those who use evidence, including policymakers and practitioners.

### Improvement of health outcomes

Therefore, the final endpoint that determines the impact of a research strategy should be the improvement of health outcomes, measured using different indicators, such as use of efficient medical techniques, improving diagnostic skills and treatments by general practitioners, health services utilisation, and accessibility, as well as those related to the patient perspective and taking into account policymakers and stakeholders [31,32].

## Conclusion

The Research Strategy indicates a global direction for research in GP/FM and serves as a basis for more detailed strategic plans in individual countries, which should take into account the characteristics of a country, its specific needs and the level of current research capacity. Those involved in GP/FM research across Europe should utilise the Research Strategy for their own country’s particular context and stage, selecting the appropriate recommendations relevant for their current situation and future direction.

## Supplementary Material

Supplementary Material

## Abbreviations

EGPRN: European General Practice Research Network
GP/FM: General Practice/Family Medicine
KTE: Knowledge transfer and exchange
PLA: Participatory Learning and Action research
RCTs: Randomised controlled trials
RCB: Research capacity building
RWE: Real World Evidence
RWD: Real World Data
WONCA: World Organisation of National Colleges, Academies and Academic Associations of General Practitioners/Family Physicians
WHO: World Health Organisation
