# Supplementary material for: The EGPRN Research Strategy for general practice in Europe
Source: Eur J Gen Pract. 2022 Jun 6;28(1):136–41. doi: 10.1080/13814788.2022.2080815 (PMC9176685; doi:10.1080/13814788.2022.2080815)
Supplement: Supplementary Material [file IGEN_A_2080815_SM7117.pdf]

EGPRN

EUROPEAN GENERAL PRACTICE
RESEARCH NETWORK

Research Strategy for General Practice in Europe 2021

Claire Collins (Editor)

Authors: Claire Collins, Esperanza Diaz, Davorina Petek, Miguel Angel Muñoz, Concepción Violán Fors, Athina Tatsioni, Sophie Eliat-Tsanani, Heidrun Lingner, Radost Assenova, Christos Lionis, Frank Dobbs, Mehmet Ungan and Victoria Tkachenko

On behalf of the EGPRN Executive Board

Contents

Executive Summary 4

Background 6

Rationale for the 2021 EGPRN Research Strategy 7

References 8

EGPRN Research Strategy 2021 11

Vision 11

Mission 11

Goals 11

Outcome Measures 12

Changes observed regarding research topics 2010-2019 14

Introduction 14

Methodology 14

Bibliographic search summary 15

Conclusion 16

References 16

ANNEX I 17

ANNEX II 28

Changes observed regarding research methodologies 2010-2019 29

Introduction 29

Research methods, research methodology and content of research 29

Methodology 29

What is new since the last RA? 30

Conclusion 31

References 31

The EGPRN Research Strategy – theory and practice 36

Theoretical Perspective 36

Relevant Actions 37

Conclusion 40

References 40

The Way Forward in Summary 41

References 41

Acknowledgements 43

2021

Research Strategy for General Practice in Europe

European General Practice Research Network

Editor: Claire Collins

Authors:

Claire Collins

Esperanza Diaz

Davorina Petek

Miguel Angel Muñoz

Concepción Violán Fors

Athina Tatsioni

Sophie Eliat-Tsanani

Heidrun Lingner

Radost Assenova

Christos Lionis

Frank Dobbs

Mehmet Ungan

Victoria Tkachenko

On behalf of the EGPRN Executive Board

Claire Collins

Thomas Frese

Jean Yves Le Reste

Ana Luisa Neves

Davorina Petek (Chair)

Pemra Unalan

Tiny van Merode

Shlomo Vinker

Graphic Design: Gözde Kemeröz Usgurlu
e-mail: gozde@uskur.com.tr

Editorial Office: Mine Kaya Bezgin, EGPRN Secretary
e-mail: office@egprn.org

EUROPEAN GENERAL PRACTICE
RESEARCH NETWORK

This publication is available on the EGPRN website; **www.egprn.org**

ISBN 9789082967388

First Published: 01 October 2021

Executive Summary

Claire Collins and Esperanza Diaz

A strong research basis is a necessity to provide effective health care and research in general practice is important in terms of improving patient outcomes effectively.

The EGPRN Executive Board considered that ten years on from its Research Agenda, it was time to review developments and consider the current landscape. The authors are the members of the EGPRN Council who volunteered to undertake this work.

In this document, we present an updated Research Agenda to be used to develop a strategic research plan for future research in general practice/family medicine (GP/FM). We present this as a basic framework to be used by those involved in research in GP/FM in individual countries adapting it to their specific situation. After careful consideration, we have moved in this instance from an 'agenda' to a 'strategy' in order to advance from presenting a series of problems and related actions to providing an overall plan with guidance to achieve specific goals.

The vision of this Research Strategy is to promote relevant research of the highest quality within GP/FM where an evidence based culture informs efforts to improve the health of citizens and to develop services.

The mission of the EGPRN Research Strategy 2021 is to contribute to the strategic development and growth of research and innovation across the European GP/FM research community. The four goals of the strategy are:

- To identify priorities for GP/FM research in order to meet needs;
- To support research capacity building;
- To develop and promote high standards of research practice;
- To foster the translations of evidence into practice.

The impact of a research strategy can be measured in terms of capacity building, scientific productivity (dissemination of research results), establishment of networks and collaborations, involvement of stakeholders and policy makers, involvement of the population and eventually in improving the health of the citizens.

Our review of publications since the 2009 EGPRN Research Agenda showed:

- The highest number of publications in the past 10 years related to the instruments and outcome measures for the competencies related to GP/FM;
- A notable number of publications dealing not just with the biopsychosocial care model or comprehensive approach, but analysing them from the point of view of their efficacy were observed;
- Shared decision making, stakeholder engagement and patients' preferences were the lowest represented topics;
- More and more, GPs are incorporating big data, via electronic medical databases, into their research, although its use is not extended to all countries yet;
- The number of clinical trials, systematic reviews and meta-analyses carried out to generate evidence in PC/GP/FM appears to have increased but is still not extensive;
- Most papers described cross-sectional, descriptive studies.

Based on our findings, we conclude that an innovative and sustainable-oriented approach is needed in GP/FM going forward. In this respect, we refer to innovation in its broadest sense in that it includes:

- New ways of reaching representative populations and including them in co-creation of research;
- New methods to gather and analyse existing data;
- The creation and use of clinical research networks that facilitate sustainable, long-term assessment of outcomes;
- New interventions to improve trajectories for patients;
- Better collaboration in primary care across disciplines and countries;
- The systematic use of electronic records, ensuring data is collected and analysed in a manner which is

ethically sound with respect for patient rights and privacy;

• Translational research specifically for primary care; and

• An increased participation of GPs and their patients in clinical trials.

We have suggested an overall theoretical model; the elements of which are:

1. Identification of needs;

2. Analysis of data;

3. Development of new methods;

4. Implementation of research: feasibility, cost-effective analyses;

5. Evaluation of interventions;

6. Scaling-up: improvement of services.

If we are to continue to improve the quantity, quality, impact and co-ordination of GP/FM research, a key concept noted here and in the previous EGPRN Research Agenda is that of capacity. Research capacity building (RCB) is critical at the individual, organisational and environmental levels. Working to improve leadership, to support the creation of a research culture in GP/FM and to increase international collaboration and networking are fundamental in this regard.

It is necessary to consider what the knowledge deficits and the relevant actions required are and to set research priorities. Ideally these should be agreed, prioritised and coordinated at a national and international level. We have mapped relevant actions to the four goals of this strategy thereby permitting the EGPRN to identify how it can support its members.

We propose that GP/FM researchers need to establish and engage in both national and international networks to ensure the successful delivery of a portfolio of high-quality studies. We need to continue to conduct primary care clinical studies dealing with common, everyday complaints and illnesses as recommended in the 2009 Research Agenda. Collaborative funding proposals on a European level should ideally be sought for such projects. However, responsible innovative approaches must be adapted to different settings and health systems, taking account of the specific context. To this end, meaningful user involvement that reflects the diversity in our communities is required. Use of existing toolkits and engagement with national and international patient platforms and representative groups are necessary. Also, knowledge transfer and exchange (KTE) is an important component to ensure a process of exchange between researchers and knowledge users in broader terms.

Background

Claire Collins, Davorina Petek,
Concepción Violán Fors and Athina Tatsioni.

The 2009 EGPRN Research Agenda

In 2009, the first European General Practice Research Network (EGPRN) Research Agenda (RA) was published[1]. It was developed at the request of WONCA Europe and related to the European Definition of General Practice/Family Medicine[2]. The agenda, for the first time, presented a proposal for European research in family medicine according to both methods and topics.

The RA summarized the then current scientific evidence related to each of the core competencies and characteristics of general practice/family medicine (GP/FM)[2], based on several key informant surveys and a comprehensive review of the scientific literature. It pointed out research needs and action points for health and research policy. A series of papers, published 2010-2011[3-8], elaborated on different aspects of the agenda focusing on topics such as the methodology used, patient centred care, problem solving skills, management and community orientation and implications for future policy and research.

The key recommendations were:

1. Better understanding and clearly defining each competency or domain (or components thereof).

2. Developing and validating instruments and outcome measures for each competency or domain (or components thereof), taking into account their complexity and interactions.

3. Developing methods of education and training for components of the different GP/FM competencies and evaluating their effectiveness, including the impact on health care and health outcomes, in the short and long term (sustainability).

4. Studying patients' and doctors' perceptions, perspectives and preferences regarding specific components or aspects of each research domain (for example: practice management issues, communication, patient involvement and choice).

5. Evaluating effectiveness and efficiency of a patient-centred approach, a comprehensive approach, a biopsychosocial care model, and community orientated healthcare (as compared to a biomedical and specialist approach), including different models or management strategies. These should be studied in populations with different cultural, social, or geographic contexts.

6. Developing primary care databases as a basic infrastructure for both health care and research, including studying and improving the utility and validity of data from electronic patient records in GP/FM.

7. Performing high quality longitudinal studies on primary care epidemiology and the development of illness over the course of time, and considering medical as well as functional and quality of life outcome measures. These studies should be based on primary care data featuring reasons for encounter as well as diagnoses, and mapping episodes of care.

8. Including data on reasons for encounter/episodes of care, as well as diagnoses, functional and quality of life outcome measures.

9. Attempting to understand how social, cultural and environmental circumstances influence health differences between populations.

10. Conducting primary care clinical studies dealing with common, everyday complaints and illnesses in non-selected GP/FM patients. Such studies should also address diagnostic reasoning (starting from complaints and symptoms and dealing with uncertainty and complexity and using step-wise strategies, including watchful waiting and assumptive treatment of symptoms, and focusing on simple or portable and point-of-care diagnostic methods) and including therapeutic trials (including comparisons of established treatments, stop-trials, safety studies and non-pharmaceutical interventions).

11. Exploring implications of multi-morbidity or curative and preventive aspects of care in the same patients.

General Practice in context

In 2011, WONCA Europe[2] summarized the central characteristics that should define the abilities of general practitioners.

1. *Primary care management*: Primary care management is a broad concept which includes access

to health care, coordination, analyses of models of care, and clinical competence issues.

2. *Community orientation:* Ability to reconcile the health needs of individual patients and the health needs of the community in balance with available resources.

3. *Person-centred care, comprehensive and holistic approach:* This includes communication skills regarding relationships with patients and families, longitudinal nature of care, and promotion, prevention and palliative measures, all together with a biopsychosocial approach.

4. *Specific problem solving skills:* This is a wide concept including clinical skills, such as diagnosis and therapy, overlapped with management.

5. *Quality improvement*

The effectiveness of any national healthcare system is strongly correlated with the strength and position of primary care within that system[9]. The international evidence overwhelmingly demonstrates that health systems oriented toward general practice and primary health care (PHC) produce better health outcomes at lower costs and with higher user satisfaction[10]. PHC is organised differently across Europe, but is typically characterised by four dimensions: access, continuity of care, coordination of care and comprehensiveness of care[11]. In most countries, the key health professional is the general practitioner (GP) or the family doctor (FD), depending on the development of the medical specialties in the country. Despite some differences in their curriculum and approach, both have the main responsibility for the treatment and management of most health problems in society at the lowest level of care[12]. They are in contact with most of the population in their country every year and play a key role in preventing disease and improving the health of the population[13]. The strengthening of primary care is widely encouraged[15]. A strong research basis is essential for a strong primary care system[15] and clinical research improves individual patient care[16]. Furthermore, the recent COVID-19 pandemic has demonstrated the need for rapid evidence development and evolution in general practice, making the need for practitioners to gain research capacity and skills to effect change paramount[17].

Rationale for the 2021 EGPRN Research Strategy

In the past ten years, there have been many changes in clinical knowledge in medicine, health services organisation, and socio-demographics connected with patient needs. These changes have challenged the validity of the World Health Organization's definition of health in the 21st century[18]. A new way of viewing human health has emerged including the ability to adapt and to self-manage[19]. This more dynamic formulation requires a different organization and delivery of services that will support patients' resilience and capacity to cope and to maintain and restore one's integrity, equilibrium, and sense of wellbeing[20]. To that end, new models of health care delivery have been developed in primary health care, including integrated primary care[21]. These primary care models need strong linkages with public health, community services, and other sectors that address the social determinants of health[22]. GP/FM interventions need to sufficiently incorporate this perspective while ensuring effectiveness, safety, and patient-centeredness.

According to the WHO Declaration of Astana[23], primary care is crucial for the health system, and enhancing the capacity and infrastructure of primary care is a key task for governments all around the world. People worldwide have different unaddressed health needs, but the burden of non-communicable disease and unhealthy lifestyles calls for integrated health services across health promotion, prevention, curative services, rehabilitation and palliative care to be accessible to all. Local communities are in a position to support and strengthen PHC. Health needs have to be responded to with a comprehensive range of services and non-fragmented care, with a good referral system and cooperation between the different levels of the health system. In order to attain these goals, a fast transfer of scientific knowledge has to be enabled. Information systems have to support the collection of data to monitor the performance of the health system according to all of the criteria for good health care[23].

Policymakers in the Astana Declaration recommitted to orienting health systems around prevention with primary health care teams as the leading source for this change[23]. However, we need to adequately define the role for GP/FM as part of the primary care team

in prevention. In addition, GP/FM is part of a wider health care spectrum, including care for acute and chronic illness as well as palliative care and care at the end of life. To inform clinical practice and policy with robust evidence, we need to evaluate the GP/FM contribution to the performance of health care services covering all relevant health needs.

Health services are responding to the new health problems of the population and a GP/FM workforce shortage[14,24-30]. Family medicine is at a critical time. Family doctors are adapting to new roles - more services, new diagnostic methods, quicker diagnosis and treatment, while some other services and community activities, especially promotion and preventive services, are increasingly offered by other professionals, such as nurses and physiotherapists. This new scenario requires the definition of a new role for family doctors and other professionals working in primary health care centres. In particular with respect to the COVID-19 pandemic, it became obvious again that "primary care remains the cornerstone of pandemic response."[31] The pandemic highlighted primary care's adaptability and how it played its part in the move to community-centred care[32].

New practice and research models, such as the Care and Learn Model[33], are proposed to help identify research gaps and improvement opportunities, evaluate existing programs, inform priority setting, and develop effective responses to the evolving needs of a rapidly changing healthcare landscape[33].

Information is increasing rapidly due to advances in technology, and this imposes new challenges on primary health care[34]. Electronic health records as well as new applications and devices provide big data repositories that promise to enhance healthcare's ability to respond to the needs of patients and populations[35]. GP/FM may have a major role in collecting and using these data in the clinical decision making process. Real World Evidence (RWE) and Real World Data (RWD) are playing an increasing role in health care decisions. The Food and Drug Administration (FDA) uses RWD and RWE to monitor post-market safety and adverse events and to make regulatory decisions. The health care community is using these data to support coverage decisions and to develop guidelines and decision support tools for use in clinical practice. Medical product developers are using RWD and RWE to support clinical trial designs (e.g., large simple trials, pragmatic clinical trials) and observational studies to generate innovative, new treatment approaches.

Previous studies have named three levels in primary health care: structure, process, and outcome[11,36]. Studies with primary data as well as meta-analysis may reveal potential strengths and weaknesses in any of these levels. Subsequent GP/FM interventions designed either to disseminate good practices or to bridge identified gaps need to be rigorously assessed for further use. In addition, evaluation may also include measuring primary health care performance indicators as appropriate[37,38]. Previous literature has also shown that improvements in structures or processes are not always translated to improvement in patient outcomes[39-41].

The EGPRN Executive Board considered that ten years on from its Research Agenda, it was time to review developments and consider the current landscape, updating the agenda as required. All members of the EGRPN Council were invited to participate in this activity. Those listed as authors self-nominated and were actively involved in writing this document. Three working groups concentrated on different aspects led by Prof. Davorina Petek, Prof. Esperanza Diaz and Dr. Miguel Angel Muñoz. Dr. Claire Collins directed the work, provided overall co-ordination of the working group leads and compiled and edited all inputs. All work was undertaken on a voluntary unpaid basis.

In this document, we present an updated Research Agenda to be used to develop a strategic research plan for future research in GP/FM. We present this as a basic framework to be used by those involved in research in GP/FM in individual countries adapting it to their specific situation, level of research capacity, medical system and organisation of health services, and specific needs of their country's population. After careful consideration and discussion among the team, we have moved in this instance from an 'agenda' to a 'strategy' in order to advance from presenting a series of problems and related actions to providing an overall plan with guidance to achieve specific goals.

References

1. Hummers-Pradier E, Beyer M, Chevallier P, et al. The Research Agenda for general practice/family medicine and primary health care in Europe. European General Practice Network (EGPRN). Maastricht, 2009.

2. WONCA Europe. The European Definition of General Practice/Family Medicine WONCA Europe 2011 Edition. Available at: http://www.woncaeurope.org/sites/default/files/documents/Definition%203rd%20ed%202011%20with%20revised%20wonca%20tree.pdf.
3. Hummers-Pradier E, Beyer M, Chevallier P, et al. The Research Agenda for General Practice/Family Medicine and Primary Health Care in Europe. Part 1. Background and methodology. *European Journal of General Practice*, 2009; 15: 243–250.
4. Hummers-Pradier E, Beyer M, Chevallier P, et al. The Research Agenda for General Practice/Family Medicine and Primary Health Care in Europe. Part 2. Results: Primary care management and community orientation. *European Journal of General Practice*, 2010; 16: 42–50.
5. Beyer M, Chevallier P, et al. The Research Agenda for General Practice/Family Medicine and Primary Health Care in Europe. Part 3. Results: Person centred care, comprehensive and holistic approach. *European Journal of General Practice*, 2010; 16: 113–119.
6. Hummers-Pradier E, Beyer M, Chevallier P, et al. The Research Agenda for General Practice/Family Medicine and Primary Health Care in Europe. Part 4. Results: Specific problem solving skills. *European Journal of General Practice*, 2010; 16: 174–181.
7. Van-Royen P, Beyer M, Chevallier P, et al. The Research Agenda for General Practice/Family Medicine and Primary Health Care in Europe. Part 5: Needs and implications for future research and policy. *European Journal of General Practice*, 2010; 16: 244–248.
8. Van-Royen P, Beyer M, Chevallier P, et al. The Research Agenda for General Practice/Family Medicine and Primary Health Care in Europe. Part 6: Reaction on commentaries – how to continue with the Research Agenda? *European Journal of General Practice*, 2011; 17: 58–61.
9. Starfield B, Shi L, Macinko J. Contribution of primary care to health systems and health. Milbank Q. 2005; 83:457–502.
10. Chan M. Return to Alma-Ata. Lancet Lond Engl. 2008 Sep 13; 372(9642): 865–6.
11. Kringos DS, Boerma WGW, Hutchinson A, et al. The breadth of primary care: a systematic literature review of its core dimensions. BMC Health Serv Res. 2010 Mar 13; 10:65.
12. Kringos DS, Boerma WGW, van der Zee J, et al. Political, cultural and economic foundations of primary care in Europe. Soc Sci Med 1982. 2013 Dec; 99: 9–17.
13. The Alma-Ata conference on primary health care. WHO Chron. 1978 Nov; 32(11): 409–30.
14. World Health Organization (WHO). The World Health Report 2008: primary health care now more than ever. 2008. Geneva, World Health Organization.
15. Beasley JW, Starfield B, van Weel C, et al. Global health and primary care research. J Am Board Fam Med. 2007;20: 518–526.
16. Mant D. R&D in primary care. National working group report. 1997. London, HMSO.
17. Tapp H. The Changing Face of Primary Care Research and Practice-Based Research Networks (PBRNs) in Light of the COVID-19 Pandemic. J Am Board Fam Med 2020; 33: 645-649.
18. World Health Organization (WHO). Constitution of the World Health Organization. 2006. www.who.int/governance/eb/who_constitution_en.pdf.
19. Huber M, Knottnerus JA, Green L, van der Horst H, et al. How should we define health? BMJ. 2011 Jul 26;343:d4163. doi: 10.1136/bmj.d4163.
20. Health Council of the Netherlands. Publication A10/04. www.gezondheidsraad.nl/sites/default/files/bijlage%20A1004_1.pdf
21. Stange KC. The problem of fragmentation and the need for integrative solutions. Ann Fam Med. 2009;7(2):100–103.
22. Gallacher K, Jani B, Morrison D, et al. Qualitative systematic reviews of treatment burden in stroke, heart failure and diabetes - methodological challenges and solutions. BMC Med Res Methodol. 2013;13:10.
23. World Health Organization (WHO). Declaration of Astana. https://www.who.int/docs/default-source/primary-health/declaration/gcphc-declaration.pdf?sfvrsn=380474fa_22.
24. OECD/European Union. Health at a Glance: Europe 2018: State of Health in the EU Cycle. Paris/European Union, Brussels: OECD Publishing; 2018. Available from: https://doi.org/10.1787/health_glance_eur-2018-en.
25. Koike S,Matsumoto S, Kodama T et al (2010) Specialty choice and physicians' career paths in Japan: an analysis of National Physician Survey data from 1996 to 2006. Health Policy 98(2):236–244.
26. López-Roig S, Pastor MÁ, Rodríguez C (2010) The reputation and professional identity of family medicine practice according to medical students: a Spanish case study. Aten Primaria 42(12):591–601.
27. Lambert T, Goldacre M (2011) Trends in doctors' early career choices for general practice in the UK: longitudinal questionnaire surveys. Br J Gen Pract 61(588):e397–e403.
28. Barber S, Brettell R, Perera-Salazar R et al (2018) UK medical students' attitudes towards their future careers and general practice: a cross-sectional survey and qualitative analysis of an Oxford cohort. BMC Med Educ 18(1):160.
29. Basu S et al (2019) Association of primary care physician supply with population mortality in the United States, 2005-2015. JAMA Intern Med 179(4):506–514.
30. Owen K et al (2019) GP retention in the UK: a worsening crisis. Findings from a cross-sectional survey. BMJ Open 9(2):e026048.
31. Health Services Executive (HSE). Medical Workforce Planning: Future Demand for General Practitioners 2015-2025. Dublin: National Doctor Training and Planning, HR Directorate, Health Service Executive (Ireland); 2015. Available from: https://www.hse.ie/eng/staff/leadership-education-development/met/plan/gp-medical-workforceplanning-report-sept-2015.pdf.
32. Rawaf S, Allen LN, Stigler FL, et al. Lessons on the COVID-19 pandemic, for and by primary care professionals worldwide. *European Journal of General Practice*, 2020; 26: 129–133.
33. Nacoti M, Ciocca A, Giupponi A, et al., At the Epicenter of the Covid-19 Pandemic and Humanitarian Crises in Italy: Changing Perspectives on Preparation and Mitigation, NEJM Catalyst March, 2020.

34. Montori VM, Hargraves I, McNellis RJ, Ganiats TG, Genevro J, Miller T, Ricciardi R, Bierman AS. The Care and Learn Model: a Practice and Research Model for Improving Healthcare Quality and Outcomes. J Gen Intern Med. 2019 Jan;34(1):154-158.

35. van der Kleij RMJJ, Kasteleyn MJ, Meijer E, Bonten TN, Houwink EJF, Teichert M, van Luenen S, Vedanthan R, Evers A, Car J, Pinnock H, Chavannes NH. SERIES: eHealth in primary care. Part 1: Concepts, conditions and challenges. Eur J Gen Pract. 2019 Oct;25(4):179-189.

36. Kruse CS, Goswamy R, Raval Y, Marawi S. Challenges and Opportunities of Big Data in Health Care: A Systematic Review. JMIR Med Inform. 2016 Nov 21;4(4):e38.

37. Kringos DS, Boerma WG, Bourgueil Y, et al. The European primary care monitor: structure, process and outcome indicators. BMC Fam Pract. 2010 Oct 27; 11:81.

38. Veillard J, Cowling K, Bitton A, et al. Better Measurement for Performance Improvement in Low- and Middle-Income Countries: The Primary Health Care Performance Initiative (PHCPI) Experience of Conceptual Framework Development and Indicator Selection. Milbank Q. 2017 Dec; 95(4): 836-883.

39. Kringos D, Nuti S, Anastasy C, et al. Re-thinking performance assessment for primary care: Opinion of the expert panel on effective ways of investing in health. Eur J Gen Pract. 2019 Jan; 25(1): 55-61.

40. Mangione CM, Gerzoff RB, Williamson DF, et al. The association between quality of care and the intensity of diabetes disease management programs. Ann Intern Med. 2006 Jul 18; 145(2): 107-116.

41. Selby JV, Swain BE, Gerzoff RB, et al. Understanding the gap between good processes of diabetes care and poor intermediate outcomes: Translating Research into Action for Diabetes (TRIAD). Med Care. 2007 Dec; 45(12): 1144-1153.

42. TRIAD Study Group. Health systems, patients factors, and quality of care for diabetes: a synthesis of findings from the TRIAD study. Diabetes Care. 2010 Apr; 33(4): 940-947.

EGPRN Research Strategy 2021

Davorina Petek, Concepción Violán Fors,
Athina Tatsioni and Claire Collins

This research strategy presents a view on how GP/FM research should develop over the next decade for those involved in all aspects of GP/FM research.

A key aim is to ensure that we produce robust and relevant evidence, to make GP/FM care more effective, safer, and more patient-centred, as well as more accessible, equitable, and affordable.

This strategy takes a global view of research in GP/FM, that is multidisciplinary and includes international collaborative research to capture the differences between countries, that is close to the users and practices, which are an important source of data, and incorporates the translation of research results into practice.

Researchers from each European country may use this strategy and adopt it to local population needs and research capacity after considering the health care services organization in their own country. Despite potential disparities, we believe that the EGPRN Research Strategy 2021 may also serve as a reference document for GP/FM researchers who may identify areas for collaborative projects among European countries.

The vision, mission and goals of the strategy were developed by the authors through discussion taking into account relevant literature and with input from the EGPRN Executive regarding its purpose.

Vision

The vision of this Research Strategy is to promote relevant research of the highest quality within general practice/family medicine where an evidence based culture informs efforts to improve the health of citizens and to develop services.

Mission

The mission of the EGPRN Research Strategy 2021 is to contribute to the strategic development and growth of research and innovation across the European GP/FM research community.

Goals

Here we will present specific goals which the strategy is intended to achieve. Stakeholders to communicate the strategy to are diverse, including but not limited to, patients/population, training organisations, philanthropists, private companies, investors, research institutions, health service providers, State Governments, Departments/Agencies of Health, other State Government agencies and policy makers and national and international funders.

Goal 1: To identify priorities for general practice/ family medicine research in order to meet needs

The identification of key methodological and subject priorities is important for research in general practice/family medicine in order to stimulate research that will help in meeting the needs of society and in informing how to develop and maintain sustainable family medicine. These should reflect the contemporary issues in family medicine and consider its future development and direction. While this strategy outlines subject and methodological areas to focus on, ongoing engagement is necessary in order that priorities reflect the needs of the discipline and its stakeholders and are relevant to different countries as they progress through their research development continuum.

Goal 2: To support research capacity building

There are multiple objectives within this goal which cross the individual, organisational and environmental levels. We recognise the need to cultivate a climate of opinion in which research in family medicine and development is expected, valued and rewarded in society. Increased partnerships with other stakeholders should be encouraged which enable collaboration across countries and between entities within countries. Further development of public and patient involvement (PPI) in research in the field of general practice/family medicine is necessary. Training for individuals and the utilization

of train the trainer and mentoring to promote knowledge acquisition in order to assist in bridging the gap in capacity across countries. Promotion of research networks within general practice/family medicine is required.

Goal 3: To develop and promote high standards of research practice

We must ensure adherence to best practice research guidelines. All those involved in research should be aware of their professional responsibilities – this requires setting standards and providing training in areas such as data protection legislation, research ethics and research integrity.

Goal 4: To foster the translation of evidence into practice

It is important to contribute to the implementation of research evidence into everyday practice by supporting knowledge translation through the synthesis of research findings, dissemination of research findings and exchange of knowledge. Efforts are necessary to enhance the adoption of research and understanding of findings by general practitioners/family physicians and to ensure that clinical guidelines are based on sound evidence. Translational outcomes (e.g. public engagement, commercialisation and implementation of interventions), health care delivery outcomes (e.g. quality and efficiency measures), policy outcomes (e.g. changes to policies, pathways and guidelines) along with the translation of discoveries into treatments and services that improve people's lives should be sought.

Outcome Measures

The global outcome measures of this research strategy aim to support the three levels of primary care (structure, process, and outcome), the importance of core competencies and their impact on quality health care, and links to other dimensions and outcomes of primary care. It is important to develop outcomes which are multidimensional and those that include factors such as patient satisfaction, health indicators and economic indicators.

This strategic document indicates a global direction for research in family medicine and serves as a basis for more detailed strategic plans in individual countries that will take into account characteristics of the country, its specific needs and its level of current research capacity and development.

A strategic plan (SP) is an organizational process used to make decisions and allocate resources to accomplish a set of goals. Once the priorities are decided and the actions made, the success of the plan must eventually be established by measuring outcomes/results.

In the case of health care, the generation of knowledge alone is not sufficient to facilitate a more productive interface between researchers and those who use evidence, including policymakers and practitioners[3]. Therefore, the final endpoint that determines the impact of a research strategy should be the improvement of health outcomes, measured using different indicators, such as use of efficient medical techniques, improving diagnostic skills and treatments by general practitioners, health services utilization, and accessibility, as well as those related to the patient perspective and taking into account policy makers and stakeholders. Research should also justify the importance of health policy management and primary care support for the health care process (regarding relevance of core competencies, values and dimensions).

The main way that health research is measured as having impact is by research findings having a measurable impact on clinical practice, health policy and behavioural change:

- better patient outcomes;
- beneficial change to health practices;
- evidence of increased efficiency in the health system;
- commercialisation of health research outcomes; and
- support for the use of and outcome from funding.

When measuring outcomes, we could use the approach of implementation science, which is the study of strategies used to integrate evidence-based practices into real-world settings[1]. Outcomes can be classified in terms of implementation, services or client (See Figure 1).

Figure 1: *Types of outcomes in implementation research*[2].

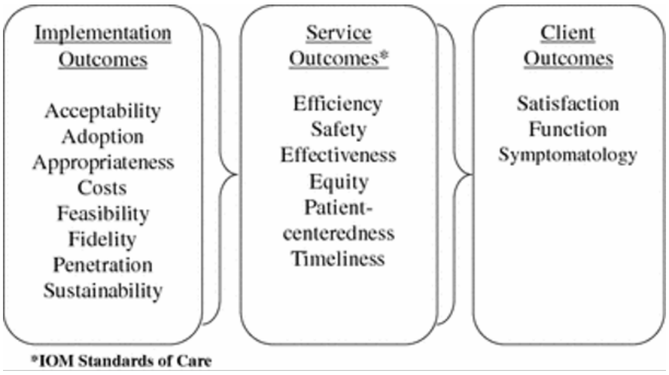

Source: Proctor E, Silmere H, Raghavan R, Hovmand P, Aarons G, Bunger A, et al. Outcomes for Implementation Research: Conceptual Distinctions, Measurement Challenges, and Research Agenda. Administration and Policy in Mental Health and Mental Health Services Research. 2011; 38: 65–76.

Outcomes must be SMART - Specific, Measurable, Achievable, Relevant and Time-bound[3].

On the other hand, it is difficult to establish how to evaluate the real impact of a research strategy on the different stakeholders involved in research. As a basic document for orientation of research, its impact is indirect and quantitative measures are subject to many biases, so other outcome measures, such as qualitative ones, might also be important.

The impact of a research strategy can be measured in terms of capacity building, scientific productivity (dissemination of research results), establishment of networks and collaborations, involvement of stakeholders and policy makers, involvement of the population and eventually in improving the health of the citizens. While recognising that other factors may also come into play, consideration should be given to how the specific impact of your strategy might be measured. However, a causal effect may not be possible to distinguish.

The EGPRN, as the author of this research strategy, has documented for itself outcome indicators to monitor in order to measure the impact of this strategy.

References

1. Lewis CC, Mettert KD, Dorsey CN, Martinez GR, et al. An updated protocol for a systematic review of implementation-related measures. Syst Rev 7, 66 (2018) doi:10.1186/s13643-018-0728-3.
2. Proctor E, Silmere H, Raghavan R, et al. Outcomes for Implementation Research: Conceptual Distinctions, Measurement Challenges, and Research Agenda. Administration and Policy in Mental Health and Mental Health Services Research. 2011; 38: 65–76.
3. World Health Organisation (WHO). Planning, Monitoring and Evaluation Framework for Research Capacity Strengthening. ESSENCE Good practice document series. 2016. WHO. https://www.who.int/tdr/publications/Essence_frwk_2016_web.pdf
4. Titler, M.G., (May 31, 2018) "Translation Research in Practice: An Introduction" *OJIN: The Online Journal of Issues in Nursing* Vol. 23, No. 2, Manuscript 1. **DOI:** 10.3912/OJIN.Vol23No02Man01

Changes observed regarding research topics 2010-2019

Miguel Angel Muñoz, Christos Lionis,
Frank Dobbs, Mehmet Ungan
and Victoria Tkachenko

Introduction

We carried out a bibliographic search to identify the changes observed in GP/GM research that parallel the recommendations of the European General Practice Research Network (EGPRN) Research Agenda (RA)[1] on GP/FM research from 2010 to 2019.

The previous RA was written using a bibliographic research methodology, based on the six core principles of GP/FM: primary care management, person-centred care, comprehensive and holistic approach, community orientation and specific problem solving skills[2].

It has been reported that, among the high number of presentations made at the various EGPRN meetings between 1999-2006, only 60% were published in English Medline-listed journals, which limits the visibility of this research[3].

We have looked broadly at research overall undertaken in this field of GP/FM over the past ten years, with a focus on the "*final recommendations for future research*" made in the 2009 RA.

Methodology

A bibliographic search was undertaken based on the final recommendations for future research made in the previous RA, using the key words considered most relevant to these recommendations.

While we are not able to attribute causation or define the direct impact of the RA on research undertaken in primary care, we can outline the changes observed in primary care research that are in line with the recommendations in the agenda.

An extensive literature search was conducted in Pub Med (see Annex I for search strategy and terms) for the period of 1 January 2010 to 31 December 2019. All searches were restricted to publications with an English language abstract available. Limits were applied to exclude publications from the areas outside Europe or EGPRN countries. As a consequence, the following limits were applied "NOT United States NOT Asia NOT China NOT Japan NOT Australia NOT New Zealand NOT India NOT Russia NOT South America NOT Africa". The following terms were applied to all searches in addition to those listed in Annex I under each topic area: Primary health care OR Family Practice OR General Practice OR Family Physician.

The recommendations related to topics from the RA outlined above in the background (specifically recommendations 2 to 6 have been grouped as a set of broad topic areas)

Recommendation	Broad topic areas
Developing and validating instruments and outcome measures for each competency or domain (or components thereof), taking into account their complexity and interactions	Primary care management<br>Patient-centred care<br>Specific problem solving competency<br>Comprehensive and holistic approach<br>Community orientation
Developing methods of education and training for components of the different GP/FM competencies and evaluating their effectiveness, including the impact on health care and health outcomes, in the short and long term (sustainability)	Patient education<br>Training methodology<br>Sustainability
Studying patients' and doctors' perceptions, perspectives and preferences regarding specific components or aspects of each research domain	Patient preferences<br>Doctor perceptions<br>Communication/Communication skills<br>Patient involvement<br>Public involvement<br>Shared decision making<br>Self-management<br>Decision support<br>Stakeholder engagement
Evaluating effectiveness and efficiency of a patient-centred approach, a comprehensive approach, a biopsychosocial care model, and community orientated healthcare including different models or management strategies	Patient-centred approach<br>Comprehensive approach<br>Biopsychosocial care<br>Community orientated healthcare<br>Integrated primary care
Developing primary care databases as a basic infrastructure for both health care and research including studying and improving the utility and validity of data from electronic patient records in GP/FM	Research based on registries

Bibliographic search summary

A full report of bibliographic findings is summarized in Annex I.

In relation to the first topic, (*"Developing and validating instruments and outcome measures for each competency or domain (or components thereof), taking into account their complexity and interactions"*), a total of 1,886,626 articles were published in the study period.

Regarding the second topic, (*"Developing methods of education and training for components of the different GP/FM competencies and evaluating their effectiveness, including the impact on health care and health outcomes, in the short and long term"*), a total of 359,368 articles were found, and among those, 45,481 were directly related to primary care/general practice/family medicine (PC/GP/FM).

"Studying patients' and doctors' perceptions, perspectives and preferences regarding specific components or aspects of each research domain" was the third topic, and 403,667 articles were found on this, with a total of 36,248 indexed publications related to PC/GP/FM.

The topic *"Evaluating effectiveness and efficiency of a patient-centred approach, a comprehensive approach, a biopsychosocial care model, and community orientated healthcare"* was associated with 162,138 articles, and after applying the filter of PC/GP/FM the number was reduced to 21,847.

Finally, there were 16,443 articles published on PC/GP/FM related to *"Developing primary care databases as a basic infrastructure for both health care and research including studying and improving the utility and validity of data from electronic patient records in GP/FM"*.

Regarding research methodology used to design the published projects, the most common were clinical trials (153,631) and reviews (124,896 articles) (full report in Annex II).

Conclusion

Among the topics analysed, the highest number of publications related to "instruments and outcome measures for each competency or domain", which includes primary care management, person-centred care, specific problem-solving competency, comprehensive approach and community orientation. Since these characteristics form the basis of primary care, this outcome is consistent with that expected.

Although the rest of the topics are less commonly approached, it is important to note the high number of publications related to patients' and doctors' perceptions, perspectives and preferences. This approach gives consideration to the feelings of both patients and GPs toward the healthcare provided in primary care, and examines the perspective of both sides, not just the professionals, and not just focusing on biology or technical aspects of care.

The high number of publications dealing not just with the biopsychosocial care model or comprehensive approach, but analysing them from the point of view of their efficacy, is also remarkable.

Shared decision making, stakeholder engagement and patients' preferences are the lowest represented topics.

More and more, GPs are incorporating big data, via electronic medical databases, into their research, although its use is not extended to all countries yet.

Finally, the number of clinical trials, systematic reviews and meta-analyses carried out to generate evidence in PC/GP/FM appears to have increased.

The bibliographic search we are presenting here has some limitations, including from the medical database itself. PubMed does not enable limiting a search to only EGPRN countries and languages and even if so, it would not be valid to suggest that the changes observed were linked to the previous EGPRN RA. Nevertheless, the search is broad enough to demonstrate that there has been a very large amount of research taking place in primary care/general practice over the past decade.

GPs should continue investigating the common clinical conditions which are the primary reason for patient visits, including effective interventions for them.

Since GPs increasingly have more information resources, such as electronic medical records, an effort is required to carry out more research using real world data. This will assist in validating the outcomes obtained in randomized clinical trials in real life. This methodology will be also valuable in observing and analysing a large number of events, comorbidities and clinical parameters in the daily work of a general practitioner.

References

1. Hummers-Pradier E, Beyer M, Chevallier P, et al. The Research Agenda for general practice/family medicine and primary health care in Europe. European General Practice Network (EGPRN). Maastricht, 2009.
2. WONCA Europe. The European Definition of General Practice/Family Medicine WONCA Europe 2011 Edition. Available at: http://www.woncaeurope.org/sites/default/files/documents/Definition%203rd%20ed%202011%20with%20revised%20wonca%20tree.pdf.
3. Van Royen P, Sandholzer H, Griffiths F, et al. Are presentations of abstracts at EGPRN meetings followed by publication? Eur J Gen Pract. 2010 Jun;16(2):100-5. doi: 10.3109/13814788.2010.482582.

ANNEX I

TOPIC 1: Developing and validating instruments and outcome measures for each competency or domain (or components thereof), taking into account their complexity and interactions.

KEYWORDS	SEARCH TERMS USED	SEARCH RESULTS
**Primary care management**		
Primary care management	Practice management OR patient care management	211,973
Primary care model	Primary Health Care	97,552
Primary care guidelines	Practice Guidelines	37,335
Primary care protocols	Clinical Protocols	55,154
Quality of life questionnaire	Quality of Life	146,439
Standard of care	Quality of Healthcare	1,549,834
Audit		
**Person-centred care**		
**Person-centred care**	Patient Centered Care	10,077
Patient-centred approach		
Patient satisfaction	Patient satisfaction	43,766
Patient empowerment		
**Specific problem solving competency**		
**Problem solving**	Problem solving	10,116
**Screening**	Diagnostic Screening Programs	1,054
Diagnostic	Diagnostic Techniques and Proce-dures	1,162,618
Prevention	Prevention and Control	228,564
Chronic care model	Long-term care	41,626
Chronic disease management	Chronic disease	175,013
Acute care	Acute diseases	122,624
Patient safety	Patient safety	59,956
Harm effects	Patient Harm OR Medical Errors OR quaternary prevention	40,190
Avoidable mortality	Mortality	296,645
Avoidable hospitalization	Hospitalization	1,216,334
Health service utilization	Health Services	407,634

**Comprehensive and holistic approach**		
Comprehensive approach	Comprehensive health care (*Subheadings*: classification, epidemiology, methods, organization and administration, standards, trends) *NOT effectiveness NOT efficiency*	17,457
Biopsychosocial model	Biopsychosocial model [title/abstract] *NOT effectiveness NOT efficiency*	388
Risk factor management	Risk Factors	336,144
Palliative care	Palliative Care	22,277
Elderly care	Aged	1,034,337
Emergency care	Emergency Medical Services	30,591
Holistic health	Holistic health	4,966
**Community orientation**		
Community	Community-Based Participatory Research OR Community Health Planning OR Community Participation *NOT effectiveness NOT efficiency*	11,977
Immunization		248,110
Public health		1,701,163
Maternity care	Maternal Health Services	8,629
Community mental health care	Community Mental Health Services	6,189

TOPIC 2: Developing methods of education and training for components of the different GP/FM competencies and evaluating their effectiveness, including the impact on health care and health outcomes, in the short and long term.

	MESH TERMS/KEYWORDS	SEARCH RESULTS
**Patient education**	Patient education	17,750
**Training methodology**	Teaching OR Education	334,122
**Sustainability**	Program Evaluation	35,715
	Competency-based education OR Clinical competence	27,830
Results of subtopics combined		359,368
Subtopics combined with primary health care OR family practice OR general practice OR family physician OR general practitioner		45,481

TOPIC 3: Studying patients' and doctors' perceptions, perspectives and preferences regarding specific components or aspects of each research domain.

	SEARCH TERMS USED	SEARCH RESULTS
**Patient preferences**	Patient Preference	9,894
**Doctor perceptions**	Perception	154,704
**Communication/Communication skills**	Communication	198,064
**Patient involvement**	Patient Participation	15,873
**Public involvement**	Community Participation	12,722
**Shared decision making**	Decision Making, Shared	5,177
**Self-management**	Self-Management	26,290
**Decision support**	Clinical Decision-Making	30,919
**Stakeholder engagement**	Stakeholder Participation	944
Results of subtopics combined		403,667
Subtopics combined with primary health care OR family practice OR general practice OR family physician OR general practitioner		36,248

TOPIC 4: Evaluating effectiveness and efficiency of a patient-centred approach, a comprehensive approach, a biopsychosocial care model, and community orientated healthcare (as compared to a biomedical and specialist approach), including different models or management strategies.

	MESH TERMS/KEYWORDS	SEARCH RESULTS
**Patient-centred approach**	Patient Centered Care	10,069
**Comprehensive approach**	Comprehensive Health Care	61,334
**Biopsychosocial care**	Biopsychosocial model (Title/ Abstract)	588
**Community orientated healthcare**	Community Health Planning OR CommunityMedicine OR Community Health Services	97,367
**Integrated primary care**	Delivery of Healthcare, Integrated	14,501
Results of subtopics combined		162,138
Results of subtopics combined with primary health care OR family practice OR general practice OR family physician OR general practitioner		56,372

TOPIC 5: Developing primary care databases as a basic infrastructure for both health care and research, including studying and improving the utility and validity of data from electronic patient records in GP/FM.

	MESH TERMS/KEYWORDS	SEARCH RESULTS
**Research based on registries**	Electronic Health Records research OR Medical Records research OR Medical Records Systems, Computerized OR Database research	137,446
Results after limits applied		16,433

Search strategy Topic 1

#1 primary health care OR family practice OR general practice OR family physician OR general practitioner **662,403**

#4 primary health care OR family practice OR general practice OR family physician OR general practitioner Abstract from 2010-2019 **294,824**

#5 Search #4 NOT United States NOT Asia NOT China NOT Japan NOT Australia NOT New Zealand NOT India NOT Russia NOT South America NOT Africa **165,756**

#6 practice management OR patient care management **1,015,391**

#9 practice management OR patient care management Abstract from 2010-2019 **373,430**

#10 Search #9 NOT United States NOT Asia NOT China NOT Japan NOT Australia NOT New Zealand NOT India NOT Russia NOT South America NOT Africa **211,973**

#11 primary health care **341,417**

#14 primary health care Abstract from 2010-2019 **179,484**

#15 Search #14 NOT United States NOT Asia NOT China NOT Japan NOT Australia NOT New Zealand NOT India NOT Russia NOT South America NOT Africa **97,552**

#16 practice guidelines **152,393**

#19 practice guidelines Abstract from 2010-2019 **58,622**

#20 Search #19 NOT United States NOT Asia NOT China NOT Japan NOT Australia NOT New Zealand NOT India NOT Russia NOT South America NOT Africa **37,335**

#21 clinical protocols **217,091**

#24 clinical protocols Abstract from 2010-2019 **90,674**

#25 Search #24 NOT United States NOT Asia NOT China NOT Japan NOT Australia NOT New Zealand NOT India NOT Russia NOT South America NOT Africa **55,154**

#26 quality of life **386,546**

#29 quality of life Abstract from 2010-2019 **231,588**

#30 Search #29 NOT United States NOT Asia NOT China NOT Japan NOT Australia NOT New Zealand NOT India NOT Russia NOT South America NOT Africa **146,439**

#31 quality of healthcare **6,892,871**

#34 quality of healthcare Abstract from 2010-2019 **2,807,553**

#35 Search #34 NOT United States NOT Asia NOT China NOT Japan NOT Australia NOT New Zealand NOT India NOT Russia NOT South America NOT Africa **1,549,834**

#36 Search #10 OR #15 OR #20 OR #25 OR #30 OR #35 **1,708,884**

#37 patient centered care **32,172**

#40 patient centered care Abstract from 2010-2019 **19,176**

#41 Search #40 NOT United States NOT Asia NOT China NOT Japan NOT Australia NOT New Zealand NOT India NOT Russia NOT South America NOT Africa **10,077**

#42 patient satisfaction **129,598**

#45 patient satisfaction Abstract from 2010-2019 **65,323**

#46 Search #45 NOT United States NOT Asia NOT China NOT Japan NOT Australia NOT New Zealand NOT India NOT Russia NOT South America NOT Africa **43,766**

#47 Search #41 OR #46 **52,228**

#48 problem solving **44,224**

#51 problem solving Abstract from 2010-2019 **16,448**

#52 Search #51 NOT United States NOT Asia NOT China NOT Japan NOT Australia NOT New Zealand NOT India NOT Russia NOT South America NOT Africa **10,116**

#53 diagnostic screening programs **3,591**

#56 diagnostic screening programs Abstract from 2010-2019 **1,947**

#57 Search #56 NOT United States NOT Asia NOT China NOT Japan NOT Australia NOT New Zealand NOT India NOT Russia NOT South America NOT Africa **1,054**

#58 diagnostic techniques and procedures **7,193,376**

#61 diagnostic techniques and procedures Abstract from 2010-2019 **2,004,228**

#62 Search #61 NOT United States NOT Asia NOT China NOT Japan NOT Australia NOT New Zealand NOT India NOT Russia NOT South America NOT Africa **1,162,618**

#63 prevention and control **1,413,982**

#66 prevention and control Abstract from 2010-2019 **477,230**

#67 Search #66 NOT United States NOT Asia NOT China NOT Japan NOT Australia NOT New Zealand NOT India NOT Russia NOT South America NOT Africa **228,564**

#68 long-term care **135,320**

#71 long-term care Abstract from 2010-2019 **67,375**

#72 Search #71 NOT United States NOT Asia NOT China NOT Japan NOT Australia NOT New Zealand NOT India NOT Russia NOT South America NOT Africa **41,626**

#73 chronic disease **726,645**

#76 chronic disease Abstract from 2010-2019 **308,479**

#77 Search #76 NOT United States NOT Asia NOT China NOT Japan NOT Australia NOT New Zealand NOT India NOT Russia NOT South America NOT Africa **175,013**

#78 acute diseases **601,054**

#81 acute diseases Abstract from 2010-2019 **214,520**

#82 Search #81 NOT United States NOT Asia NOT China NOT Japan NOT Australia NOT New Zealand NOT India NOT Russia NOT South America NOT Africa **122,624**

#83 patient safety **154,980**

#86 patient safety Abstract from 2010-2019 **94,857**

#87 Search #86 NOT United States NOT Asia NOT China NOT Japan NOT Australia NOT New Zealand NOT India NOT Russia NOT South America NOT Africa **59,956**

#88 patient harm OR medical errors OR quaternary prevention **157,353**

#91 patient harm OR medical errors OR quaternary prevention **63,875**

#92 Search #91 NOT United States NOT Asia NOT China NOT Japan NOT Australia NOT New Zealand NOT India NOT Russia NOT South America NOT Africa **40,190**

#93 mortality **1,240,784**

#96 mortality Abstract from 2010-2019 **551,977**

#97 Search #96 NOT United States NOT Asia NOT China NOT Japan NOT Australia NOT New Zealand NOT India NOT Russia NOT South America NOT Africa **296,645**

#98 hospitalization **5,103,080**

#101 hospitalization Abstract from 2010-2019 **2,359,280**

#102 Search #101 NOT United States NOT Asia NOT China NOT Japan NOT Australia NOT New Zealand NOT India NOT Russia NOT South America NOT Africa **1,216,334**

#103 health services **2,420,576**

#106 health services Abstract from 2010-2019 **768,209**

#107 Search #106 NOT United States NOT Asia NOT China NOT Japan NOT Australia NOT New Zealand NOT India NOT Russia NOT South America NOT Africa **407,634**

#108 Search #52 OR #57 OR #62 OR #67 OR #72 OR #77 OR #82 OR #87 OR #92 OR #97 OR #102 OR #107 **2,455,051**

#109 comprehensive health care [MeSH Major Topic] NOT effectiveness NOT efficiency **124,308**

#112 comprehensive health care [MeSH Major Topic] NOT effectiveness NOT efficiency Abstract from 2010-2019 **31,734**

#113 Search #112 NOT United States NOT Asia NOT China NOT Japan NOT Australia NOT New Zealand NOT India NOT Russia NOT South America NOT Africa **17,457**

#114 biopsychosocial model [Title/Abstract] NOT effectiveness NOT efficiency **1,157**

#117 biopsychosocial model [Title/Abstract] NOT effectiveness NOT efficiency Abstract from 2010-2019 **575**

#118 Search #117 NOT United States NOT Asia NOT China NOT Japan NOT Australia NOT New Zealand NOT India NOT Russia NOT South America NOT Africa **388**

#119 risk factors **1,292,544**

#122 risk factors Abstract from 2010-2019 **652,100**

#123 Search #122 NOT United States NOT Asia NOT China NOT Japan NOT Australia NOT New Zealand NOT India NOT Russia NOT South America NOT Africa **336,144**

#124 palliative care **83,245**

#127 palliative care Abstract from 2010-2019 **34,887**

#128 Search #127 NOT United States NOT Asia NOT China NOT Japan NOT Australia NOT New Zealand NOT India NOT Russia NOT South America NOT Africa **22,277**

#129 aged **5,265,859**

#132 aged Abstract from 2010-2019 **1,853,998**

#133 Search #132 NOT United States NOT Asia NOT China NOT Japan NOT Australia NOT New Zealand NOT India NOT Russia NOT South America NOT Africa **1,034,337**

#134 emergency medical services **152,882**

#137 emergency medical services Abstract from 2010-2019 **57,649**

#138 Search #137 NOT United States NOT Asia NOT China NOT Japan NOT Australia NOT New Zealand NOT India NOT Russia NOT South America NOT Africa **30,591**

#139 holistic health **19,802**

#142 holistic health Abstract from 2010-2019 **8,502**

#143 Search #142 NOT United States NOT Asia NOT China NOT Japan NOT Australia NOT New Zealand

NOT India NOT Russia NOT South America NOT Africa **4,966**

#144 Search #113 OR #118 OR #123 OR #128 OR #133 OR #138 OR #143 **1,246,433**

#145 (community-based participatory research OR community health planning OR community participation) NOT effectiveness NOT efficiency **71,215**

#148 (community-based participatory research OR community health planning OR community participation) NOT effectiveness NOT efficiency Abstract from 2010-2019 **27,086**

#149 Search #148 NOT United States NOT Asia NOT China NOT Japan NOT Australia NOT New Zealand NOT India NOT Russia NOT South America NOT Africa **11,977**

#150 immunization OR vaccination **1,345,762**

#153 Immunization OR vaccination Abstract from 2010-2019 **508,128**

#154 Search #153 NOT United States NOT Asia NOT China NOT Japan NOT Australia NOT New Zealand NOT India NOT Russia NOT South America NOT Africa **248,110**

#155 public health **8,098,261**

#158 public health Abstract from 2010-2019 **3,208,189**

#159 Search #158 NOT United States NOT Asia NOT China NOT Japan NOT Australia NOT New Zealand NOT India NOT Russia NOT South America NOT Africa **1,701,163**

#160 maternal health services **64,092**

#163 maternal health services Abstract from 2010-2019 **25,066**

#164 Search #163 NOT United States NOT Asia NOT China NOT Japan NOT Australia NOT New Zealand NOT India NOT Russia NOT South America NOT Africa **8,629**

#165 community mental health services **38,992**

#168 community mental health services Abstract from 2010-2019 **13,753**

#169 Search #168 NOT United States NOT Asia NOT China NOT Japan NOT Australia NOT New Zealand NOT India NOT Russia NOT South America NOT Africa **6,189**

#170 Search #149 OR #154 OR #159 OR #164 OR #169 **1,886,626**

#171 Search #36 OR #47 **1,392,988**

#172 Search #108 OR #144 **2,729,778**

Due to server error repeatedly due to volume of results, it was decided that we had to proceed with search as follows.

#173 Search #36 AND #5 **216,874**

#174 Search #47 AND #5 **144,935**

#175 Search #108 AND #5 **578,426**

#176 Search #144 AND #5 **325,740**

#177 Search #170 AND #5 **456,111**

#178 Search #173 OR #174 OR #175 OR #176 OR #177 **1,008,543**

LIMITS OF STUDY TYPE APPLIED

#179 Search #173 OR #174 OR #175 OR #176 OR #177 Clinical Trial **79,790**

#180 Search #173 OR #174 OR #175 OR #176 OR #177 Meta-Analysis **12,241**

#181 Search #173 OR #174 OR #175 OR #176 OR #177 Randomized Controlled Trial **52,816**

#182 Search #173 OR #174 OR #175 OR #176 OR #177 Review **80,903**

#183 Search #173 OR #174 OR #175 OR #176 OR #177 Systematic Reviews **18,298**

#184 Search #173 OR #174 OR #175 OR #176 OR #177 Observational Study **14,427**

Search strategy Topic 2

#1 primary health care OR family practice OR general practice OR family physician OR general practitioner **661,138**

#4 primary health care OR family practice OR general practice OR family physician OR general practitioner Abstract from 2010-2019 **294,638**

#5 Search #4 NOT United States NOT Asia NOT China NOT Japan NOT Australia NOT New Zealand NOT India NOT Russia NOT South America NOT Africa **165,846**

#7 patient education **103,093**

#10 patient education Abstract from 2010-2019 **29,924**

#11 Search #10 NOT United States NOT Asia NOT China NOT Japan NOT Australia NOT New Zealand NOT India NOT Russia NOT South America NOT Africa **17,750**

#12 teaching OR education **1,722,464**

#15 teaching OR education Abstract from 2010-2019 **757,036**

#16 Search #15 NOT United States NOT Asia NOT China NOT Japan NOT Australia NOT New Zealand NOT India NOT Russia NOT South America NOT Africa **334,122**

#17 program evaluation **157,257**

#21 program evaluation Abstract from 2010-2019 **73,738**

#22 Search #21 NOT United States NOT Asia NOT China NOT Japan NOT Australia NOT New Zealand NOT India NOT Russia NOT South America NOT Africa **35,715**

#23 competency-based education OR clinical competence **111,800**

#26 competency-based education OR clinical competence Abstract from 2010-2019 **44,903**

#27 Search #26 NOT United States NOT Asia NOT China NOT Japan NOT Australia NOT New Zealand NOT India NOT Russia NOT South America NOT Africa **27,830**

#28 Search #11 OR #16 OR#22 OR#27 **359,368**

#29 Search #5 AND #28 **45,481**

LIMITS OF STUDY TYPE APPLIED

#30 Search #5 AND #28 Clinical Trial **4,022**

#31 Search #5 AND #28 Meta-Analysis **624**

#33 Search #5 AND #28 Randomized Controlled Trial **3,601**

#34 Search #5 AND #28 Review **5,385**

#35 Search #5 AND #28 Systematic Reviews **1,868**

#36 Search #5 AND #28 Observational Study **850**

Search strategy Topic 3

#1 primary health care OR family practice OR general practice OR family physician OR general practitioner **661,774**

#4 primary health care OR family practice OR general practice OR family physician OR general practitioner Abstract from 2010-2019 **294,764**

#5 Search #4 NOT United States NOT Asia NOT China NOT Japan NOT Australia NOT New Zealand NOT India NOT Russia NOT South America NOT Africa **165,776**

#6 perception AND patient **170,133**

#9 perception AND patient Abstract from 2010-2019 **84,180**

#10 Search #9 NOT United States NOT Asia NOT China NOT Japan NOT Australia NOT New Zealand NOT India NOT Russia NOT South America NOT Africa **53,737**

#11 patient preference **24,623**

#14 patient preference Abstract from 2010-2019 **16,079**

#15 Search #14 NOT United States NOT Asia NOT China NOT Japan NOT Australia NOT New Zealand NOT India NOT Russia NOT South America NOT Africa **9,894**

#16 perception **615,515**

#19 perception Abstract from 2010-2019 **259,715**

#20 Search #19 NOT United States NOT Asia NOT China NOT Japan NOT Australia NOT New Zealand NOT India NOT Russia NOT South America NOT Africa **154,704**

#21 communication **841,715**

#24 communication Abstract from 2010-2019 **371,479**

#25 Search #24 NOT United States NOT Asia NOT China NOT Japan NOT Australia NOT New Zealand NOT India NOT Russia NOT South America NOT Africa **198,064**

#26 community participation **62,752**

#29 community participation Abstract from 2010-2019 **26,154**

#30 Search #29 NOT United States NOT Asia NOT China NOT Japan NOT Australia NOT New Zealand NOT India NOT Russia NOT South America NOT Africa **12,722**

#31 patient participation **54,444**

#34 patient participation Abstract from 2010-2019 **26,584**

#35 Search #34 NOT United States NOT Asia NOT China NOT Japan NOT Australia NOT New Zealand NOT India NOT Russia NOT South America NOT Africa **15,873**

#36 decision making, shared **11,353**

#39 decision making, shared Abstract from 2010-2019 **8,267**

#40 Search #39 NOT United States NOT Asia NOT China NOT Japan NOT Australia NOT New Zealand NOT India NOT Russia NOT South America NOT Africa **5,177**

#41 self-management **75,390**

#44 self-management Abstract from 2010-2019 **47,653**

#45 Search #44 NOT United States NOT Asia NOT China NOT Japan NOT Australia NOT New Zealand NOT India NOT Russia NOT South America NOT Africa **26,290**

#46 clinical decision-making **81,065**

#49 clinical decision-making Abstract from 2010-2019 **48,357**

#50 Search #49 NOT United States NOT Asia NOT China NOT Japan NOT Australia NOT New Zealand NOT India NOT Russia NOT South America NOT Africa **30,919**

#51 stakeholder participation **2,350**

#54 stakeholder participation Abstract from 2010-2019 **1,889**

#55 Search #54 NOT United States NOT Asia NOT China NOT Japan NOT Australia NOT New Zealand NOT India NOT Russia NOT South America NOT Africa **944**

#56 Search #10 OR #15 OR #20 OR #25 OR #30 OR #35 OR #40 OR #45 OR #50 OR #55 **403,667**

#58 Search #56 AND #5 **36,248**

LIMITS OF STUDY TYPE APPLIED

#59 Search #56 AND #5 Randomized Controlled Trial **2,698**

#61 Search #56 AND #5 Systematic Review **1,544**

#62 Search #56 AND #5 Meta-Analysis **449**

#63 Search #56 AND #5 Clinical Trial **3,008**

#64 Search #56 AND #5 Review **4,620**

#65 Search #56 AND #5 Observational Study **656**

Search strategy Topic 4

#1 primary health care OR family practice OR general practice OR family physician OR general practitioner **661,777**

#4 primary health care OR family practice OR general practice OR family physician OR general practitioner Abstract from 2010-2019 **294,764**

#5 Search #4 NOT United States NOT Asia NOT China NOT Japan NOT Australia NOT New Zealand NOT India NOT Russia NOT South America NOT Africa **165,776**

#6 patient centered care **32,138**

#10 patient centered care Abstract from 2010-2019 **19,162**

#11 Search #10 NOT United States NOT Asia NOT China NOT Japan NOT Australia NOT New Zealand NOT India NOT Russia NOT South America NOT Africa **10,069**

#12 comprehensive health care **340,424**

#15 comprehensive health care Abstract from 2010-2019 **113,822**

#17 Search #15 NOT United States NOT Asia NOT China NOT Japan NOT Australia NOT New Zealand NOT India NOT Russia NOT South America NOT Africa **61,334**

#18 biopsychosocial model [Title/Abstract] **1,644**

#21 biopsychosocial model [Title/Abstract] Abstract from 2010-2019 **889**

#22 Search #21 NOT United States NOT Asia NOT China NOT Japan NOT Australia NOT New Zealand NOT India NOT Russia NOT South America NOT Africa **588**

#23 community health planning OR community medicine OR community health services **567,209**

#26 community health planning OR community medicine OR community health services Abstract from 2010-2019 **229,401**

#27 Search #26 NOT United States NOT Asia NOT China NOT Japan NOT Australia NOT New Zealand NOT India NOT Russia NOT South America NOT Africa **97,367**

#28 delivery of healthcare, integrated **58,193**

#31 delivery of healthcare, integrated Abstract from 2010-2019 **30,480**

#32 Search delivery of healthcare, integrated NOT United States NOT Asia NOT China NOT Japan NOT Australia NOT New Zealand NOT India NOT Russia NOT South America NOT Africa 14,501

#33 Search #11 OR #17 OR #22 OR #27 OR #32 **162,138**

#34 Search #33 and #5 **56,372**

#35 Search #34 AND (effectiveness OR efficiency) **21,847**

#36 Search #34 AND effectiveness OR efficiency **986,017**

LIMITS OF STUDY TYPE APPLIED

#37 Search #34 AND (effectiveness or efficiency) Clinical Trial **3,486**

#38 Search #34 AND (effectiveness OR efficiency) Meta-Analysis **622**

#39 Search #34 AND (effectiveness OR efficiency) Randomized Controlled Trial **3,128**

#40 Search #34 AND (effectiveness OR efficiency) Review **3,540**

#41 Search #34 AND (effectiveness OR efficiency) Systematic Reviews **1,415**

#42 Search #34 AND (effectiveness OR efficiency) Observational Study **674**

Search strategy Topic 5

#1 primary health care OR family practice OR general practice OR family physician OR general practitioner **661,777**

#4 primary health care OR family practice OR general practice OR family physician OR general practitioner Abstract from 2010-2019 **294,764**

#5 Search #4 NOT United States NOT Asia NOT China NOT Japan NOT Australia NOT New Zealand NOT India NOT Russia NOT South America NOT Africa **165,776**

#6 electronic health records research OR medical records research OR medical records systems, computerized OR database research **427,473**

#9 electronic health records research OR medical records research OR medical records systems, computerized OR database research Abstract from 2010-2019 **277,997**

#10 Search #9 NOT United States NOT Asia NOT China NOT Japan NOT Australia NOT New Zealand NOT India NOT Russia NOT South America NOT Africa **137,446**

#11 Search #5 AND #10 **16,433**

LIMITS OF STUDY TYPE APPLIED

#12 Search #5 AND #10 Clinical Trial **581**

#13 Search #5 AND #10 Meta-Analysis **1,409**

#14 Search #5 AND #10 Randomized Controlled Trial **501**

#15 Search #5 AND #10 Review **3,956**

#16 Search #5 AND #10 Systematic Reviews **3,367**

#17 Search #5 AND #10 Observational Study **590**

Context for PubMed search

At the time of searching there was a new version of PubMed available as well as an older/Legacy version. We did an initial trial search to see results and based on the higher numbers available in the new PubMed version, this was selected as the database to employ for the full searches.

However, as this was a new version we did experience a few glitches when performing literature searches. These included:

- Date filter was not as easily applicable as the Legacy version. In Legacy you could simply enter for example 01/01/2010 -31/12/2019. In the new version it appeared in graph format for the entire timeline available so to apply filter you had to move the filter up the timeline, this defaulted to the current year each time so we had to keep bringing the selection back to 2019 to cover our search criteria of 2010-2019. We ensured this was applied throughout.
- Numbers seemed to vary when even applying the initial /first set of primary health care OR family practice ... etc. but numbers seemed to vary both upwards and downwards and we do not have an explanation for this. We took each "main" set as it appeared on the day and recorded those as the search result.
- When applying limits for study type you had to select and apply the study type limit twice i.e. two separate times or otherwise it would give you a result of 0 e.g. so you had Set Number and then applied limit for example of Systematic Review but first time it would not take the limit and you had to re-enter the limit in order for it to apply. We noticed this glitch and ensured we applied it twice correctly across every aspect of the searches.
- Other issues arose when conducting Search strategy for Topic 1. The search results were so large when combining sets that the database timed out. We adapted our search as per strategy outlined.

Limits applied to the 5 search topics:

- Date range 2010 – 2019
- Abstract (English)
- NOT United States NOT Asia NOT China NOT Japan NOT Australia NOT New Zealand NOT India NOT Russia NOT South America NOT Africa

ANNEX II

Methodology used in the articles (based on the Pub Med classification).

	CLINICAL TRIAL	RANDOMIZED CONTROLLED TRIAL	REVIEW	SYSTEMATIC REVIEWS	METAANALYSIS	OBSERVATIONAL STUDY
TOPIC 1 [a]	79,790	52,816	80,903	18,298	12,241	14,427
TOPIC 2 [b]	4,022	3,601	5,385	1,868	624	850
TOPIC 3 [c]	3,008	2,698	4,620	1,544	449	656
TOPIC 4 [d]	3,486	3,128	3,540	1,415	622	674
TOPIC 5 [e]	581	501	3,956	3,367	1,409	590

[a] Developing and validating instruments and outcome measures for each competency or domain (or components thereof), taking into account their complexity and interactions

[b] Developing methods of education and training for components of the different GP/FM competencies and evaluating their effectiveness, including the impact on health care and health outcomes, in the short and long term

[c] Studying patients' and doctors' perceptions, perspectives and preferences regarding specific components or aspects of each research domain

[d] Evaluating effectiveness and efficiency of a patient-centred approach, a comprehensive approach, a biopsychosocial care model, and community orientated healthcare (as compared to a biomedical and specialist approach), including different models or management strategies

[e] Developing primary care databases as a basic infrastructure for both health care and research including studying and improving the utility and validity of data from electronic patient records in GP/FM.

Changes observed regarding research methodologies 2010-2019

Esperanza Diaz, Sophie Eliat-Tsanani,
Heidrun Lingner, Radost Assenova

Introduction

In this chapter we focus on *Methodology* in a broad sense to explain **how research is done** rather than in the specific methods to be used or the content to be addressed. From this perspective, our aim here is to provide an updated overview of methodologies to be added, and/or methodologies that should be focused upon going forward. With this in mind, we will first summarise what was proposed in the previous RA[1], then briefly address the classical understanding of research methodology in general practice/family medicine and what might be some of the trends during the last 10 years from different perspectives.

Research methods, research methodology and content of research

Research methods are comprised of the **tools, strategies or techniques** that are used in research. Research in GP/FM has evolved to acknowledge the different quantitative and qualitative approaches and understand them as complementary, and there are good examples, although not as many as desired and not equally distributed in Europe, of longitudinal studies, comparative research and intervention trials, all of them suggested in the previous RA.

Methodology, as expressed above, refers to the study of **how research is done**. It entails how we find out about procedures and the manner in which knowledge is gained. Methodology outlines the principles that guide research practices. Related to this, the previous RA uses terms such as pragmatic studies, patient involvement, translational research and the creation of ethical boards and research infrastructure.

Furthermore, when looking at the previous RA, some of the recommendations could be classified as dealing with the **content of research** (what should be researched), specifically mentioned in the previous RA when recommending evaluating generic measures, or research dealing with specific diagnostic strategies and reasoning.

Methodology

Although a review of the literature regarding the methodology of research in primary health care (PHC) during the last 10 years was out of the scope of our work package, we have rapidly reviewed the achievements in the field of research in GP/FM from two points of view:

1. Research in last decade – the most cited articles.

2. Research in general practice as presented at one of the last EGPRN meetings.

Research in GP/FM 2010-2019 – a rapid overview

The most cited articles

We aimed to explore the research subject and the methods used in the papers with most impact in the field since the last strategy was published. For this purpose, the Web of Science was searched for the most cited articles using the terms Family Medicine or General practice or Primary Health Care as the main subject, excluding editorials, filtered for the period 2010-2019. Table 1 shows the top 10 papers for the last 10 years: five of them were cross sectional studies in the form of surveys, observed behaviour and use of registers, two were reviews of literature, one presented cost-effectiveness analyses using review of the literature and two papers described the effect of interventions, one of them using a RCT and the other one relying on the evaluation of a group with no control. Thematically, most of the papers dealt with clinical topics, followed by papers studying the medical encounter and Family Medicine as a profession. Table 2 shows the ten most cited studies during the last five years (2015-2019), where at least as many papers were related to the medical encounter and Family Medicine as a profession, including workload and burnout. In this list, there is also one paper dealing specifically with research methods. Interestingly, only three papers were published in a level 2 journal (all in the British Journal

of General Practice), and the number of citations of the most cited papers was under 200 over 10 years.

Presentations in EGPRN meeting May 2019

For the sake of parsimony, we randomly selected a third of the studies presented at the 88th EGPRN Meeting, Tampere - Finland, 9-12 May 2019[2]. We included all types of presentations. The theme of this meeting was multi-morbidity, a typical theme in GP/FM, and thus it was overrepresented in the presentations. Of the 28 abstracts:

- More than half were descriptive studies using both qualitative and quantitative methods;
- Five abstracts had identification of needs as the main objective;
- Four evaluated interventions;
- Two considered feasibility of the interventions to be evaluated;
- Three evaluated or validated new methods or tools;
- One abstract specifically studied research culture in GP/FM;
- None of the abstracts described the scaling-up of a previously tested intervention;
- Most studies gathered data from users (either patients or doctors);
- One paper described the participants' evaluation of an intervention.

From the above searches, we may conclude that descriptive and cross-sectional studies using both qualitative and quantitative techniques are the most common in GP/FM research. The identification of areas or problems that need to be further studied and the validation of instruments seem to be relatively well covered in our search. We found less evidence of clinical research in terms of interventions, with few RCTs and even fewer studies regarding feasibility, efficiency or efficacy. User involvement in a broad way, apart from their participation as study subject, was seldom reported. A relatively high proportion of research deals with topics related to GP/FM as a discipline and with general practitioners themselves (burnout, workload).

A broad range of research methods was used in the most cited articles and the EGPRN abstracts and some aimed to identify needs or to develop new methods. Most papers were nevertheless cross-sectional, descriptive studies. A few papers described the effect of a health intervention and one studied its efficiency. However, description and detailed implementation of interventions, feasibility studies or studies describing challenges and solutions for the implementation of interventions at a larger scale could not be identified in either the most cited papers or the EGPRN abstracts despite the RA's recommendation on increasing translational research.

What is new since the last RA?

Need new approaches to address the gaps between knowledge and practice

The implementation gaps between evidence-informed interventions and their delivery to all people everywhere were highlighted in 2019 in the 'New global Research Agenda to advance PHC and achieve universal health coverage', funded by the Bill & Melinda Gates Foundation[3,4]. This agenda was most relevant for low and middle-income countries (LMIC), but its main conclusions may be applicable to European settings. The identified gaps included:

- How to better measure PHC to identify and address challenges and
- How to adapt and scale effective interventions to bridge three main transitions:
  - evidence to policy,
  - policy to implementation and
  - implementation to system quality and its maintenance.

Following the identification of the gaps in PHC in LMIC, the research team suggested a new Research Agenda for LMIC that focuses on four key areas: organisation and models of care; quality, safety, and performance management; policy and governance; and financing of primary care systems. According to our experience in research and as members of EGPRN over a number of years, some of these seem to be relevant in many European countries. However, the relationships between research, policy and practice in primary care are complex and might not be possible to disentangle and solve without an innovative framework and theoretical understanding. To address these gaps, a number

of strategies are proposed for researchers. These include more user involvement, including patients, health care providers, policymakers and other relevant stakeholders, and the use of participatory methods.

Responsible innovation: more than technology

Innovation is a key word in research nowadays, and innovative approaches are needed to attract funding for research in many countries. The number of papers in PubMed using the terms Innovative and Family Medicine has increased in the last 10 years from 124 in 2010 to 504 in 2019. Innovation, however, is too often thought of as technological solutions, which may or may not be the priorities of patients and health providers in primary care. The UK Framework for Responsible Innovation[5] offers another approach, understanding innovation as a process that seeks to promote creativity and opportunities for science and innovation that are socially desirable and undertaken in the public interest. This approach also considers patient and public involvement in research as key to delivering creative, desirable and sustainable solutions.

Following innovation and the development of technology in other fields of knowledge, the increasing possibilities of gathering enormous amount of data (big data) offer huge possibilities for research but also raise ethical questions and increase the dangers of misusing these data for aims other than those the public is aware of. In line with this, the General Data Protection Regulation (GDPR) was approved in Europe in 2016, and became enforceable in May 2018[6]. This regulation of EU law aims to give control to individuals over their personal data and to simplify the regulatory environment for the sharing of data within and outside Europe.

Conclusion

We did not intend in our approach to undertake formal systematic reviews, and we acknowledge that the depth and breadth of this work could be much wider. However, based on our search findings and literature on developments since the RA was written, we conclude that an innovative and sustainable-oriented approach is needed in GP/FM going forward. In this respect, we refer to innovation in its broadest sense in that it includes:

- new ways of reaching representative populations and including them in co-creation of research;
- new mechanisms to gather and analyse existing data;
- the creation and use of clinical research networks that facilitate sustainable, long-term assessment of outcomes;
- new interventions to improve trajectories for patients;
- better collaboration in primary care across disciplines and countries;
- the systematic use of electronic records in an ethically sound manner;
- translational research specifically for primary care; and
- an increased participation of GPs and their patients in clinical trials.

References

1. Hummers-Pradier E, Beyer M, Chevallier P, et al. The Research Agenda for general practice/family medicine and primary health care in Europe. European General Practice Network (EGPRN). Maastricht, 2009.
2. European General Practice Research Network. 88th EGPRN Meeting, Tampere - Finland, 9-12 May 2019. www.egprn.org.
3. https://www.ariadnelabs.org/resources/articles/news/a-new-global-research-agenda-to-advance-primary-health-care-and-achieve-uhc/
4. Hirschhorn LR, Langlois EV, Bitton A, Ghaffar A. What kind of evidence do we need to strengthen primary healthcare in the 21st century? BMJ Glob Health. 2019;4(Suppl 8):e001668.
5. Council EaPSR. Framework for Responsible Innovation 2017. https://www.epsrc.ac.uk/research/framework/.
6. General Data Protection Regulation (EU) 2016/679. https://gdpr.eu

Table 1. Most cited papers in Primary Care/general Practice 2010-2019.

No.	Title	Journal (level)	Theme	Method	Number of citations
1	Effectiveness of empathy in general practice: a systematic review	BRITISH JOURNAL OF GENERAL PRACTICE (2)	Medical encounter	Systematic review	187
2	Adaptation and validation of the Charlson Index for Read/OXMIS coded database	BMC FAMILY PRACTICE (1)	Clinical	Instrument validation	181
3	General Practice and Primary Health Care in Denmark	JOURNAL OF THE AMERICAN BOARD OF FAMILY MEDICINE (1)	Family Medicine	Descriptive paper	173
4	A Method for Estimating Relative Complexity of Ambulatory Care	ANNALS OF FAMILY MEDICINE (1)	Medical encounter	Data analysis	145
5	The strength of primary care in Europe: an international comparative study	BRITISH JOURNAL OF GENERAL PRACTICE (2)	Family Medicine	Cross-sectional, descriptive	125
6	Deprescribing medication in very elderly patients with multimorbidity: the view of Dutch GPs. A qualitative study	BMC FAMILY PRACTICE (1)	Clinical	Qualitative	111
7	Point-of-Care C-Reactive Protein Testing and Antibiotic Prescribing for Respiratory Tract Infections	BMC FAMILY PRACTICE (1)	Clinical	RCT	108
8	Are you SURE? Assessing patient decisional conflict with a 4-item screening test	CANADIAN FAMILY PHYSICIAN (1)	Clinical/ Medical Encounter	Instrument validation	106
9	Predicting the risk of Chronic Kidney Disease in Men and Women in England and Wales: prospective derivation and external validation of the QKidney (R) Scores	BMC FAMILY PRACTICE (1)	Clinical	Data analysis	101
10	Barriers to GPs' use of evidence-based medicine: a systematic review	BRITISH JOURNAL OF GENERAL PRACTICE (2)	Medical encounter	Systematic review	84

Table 2. Most cited papers in Primary Care/general Practice 2015-2019.

No.	Title	Journal	Theme	Method	Number of citations
1	Tethered to the EHR: Primary Care Physician Workload Assessment Using EHR Event Log Data and TimeMotion Observations	ANNALS OF FAMILY MEDI-CINE	PC workload	Data analysis	75
2	More Comprehensive Care Among Family Physicians is Associated with Lower Costs and Fewer Hospitalizations	ANNALS OF FAMILY MEDICINE	Patient's care	Data analysis	72
3	Multiple physical and mental health comorbidity in adults with intellectual disabilities: population-based cross-sectional analysis	BMC FAMILY PRACTICE	Clinical	Data analysis	67
4	A systematic review of parent and clinician views and perceptions that influence prescribing decisions in relation to acute childhood infections in primary care	SCANDINAVIAN JOURNAL OF PRIMARY HEALTH CARE	Clinical / Medical Encounter	Systematic review	56
5	Predictors and Outcomes of Burn-out in Primary Care Physicians	JOURNAL OF PRIMARY CARE AND COM-MUNITY HEALTH	GP's feelings	Data analysis	54
6	Inappropriate antibiotic prescription for respiratory tract indications: most prominent in adult patients	FAMILY PRACTICE	Clinical	Data analysis	52
7	Persistent problems 1 year after mild traumatic brain injury: a longitudinal population study in New Zealand	BRITISH JOURNAL OF GENERAL PRACTICE	Clinical	Data analysis	49
8	Help seeking for cancer 'alarm' symptoms: a qualitative interview study of primary care patients in the UK	BRITISH JOURNAL OF GENERAL PRACTICE	Clinical	Qualitative	43
9	Series: Practical guidance to qualitative research. Part 4: Trustworthiness and publishing		Research	Descriptive	39
10	Antibiotic prescribing and patient satisfaction in primary care in England: cross-sectional analysis of national patient survey data and prescribing data	BRITISH JOURNAL OF GENERAL PRACTICE	Patients' satisfaction	Data analysis	39

Table 3. A selected third of the abstracts presented at the EGPRN meeting in Tampere, 2019 (Theme for the conference was multi-morbidity).

Title	Methodology	User involvement
Barriers and Enablers to Deprescribing in Older Patients With Multimorbidity and Polypharmacy	Needs identification	Participants
Multimorbidity and Polypharmacy in Canada: Examining Prevalence and Patterns in Primary Health Care Using a National Electronic Medical Record Database	Analysis	None
Multimorbidity in primary care: Interdisciplinary person centred disease management	Needs identification	Participants
Complex Multimorbidity - Prevalence and Workload	Analysis	None
Further development and validation of the Multimorbidity Treatment Burden Questionnaire	Development methods	Participants
INterprofessional follow-up of PATIents with Cancer (SINPATIC study): an exploratory study of patients	Needs identification Interdisci-plinarity	Participants
Anticholinergic burden and most common anticholinergic-acting medications in older general practice patients	Analysis	Participants
Identifying high-need patients with multimorbidity based on their primary care medical records	Analysis	None
Multimorbidity or dual pattern of diseases among Negev Bedouins? Approach to multimorbidity in communities under transition	Analysis	None
Bringing together older multimorbid patients with polypharmacy, general practitioners, and eHealth: protocol of a cluster randomized controlled trial in Swiss primary care	Evaluation	Participants
Measuring needs and expectations in life when living with mul-timorbidity - development and validation of the MMQoL scale	Development methods	Participants
Quality of life among older adults living with multimorbidity: findings from the European SHARE database	Analysis	Participants
Feasibility of a checklist in treating hypertension in primary care – base line results from a cluster-randomised controlled trial (check and support)	Implementation	Participants
The effect of electronic reminders on the recording of diagnoses in primary care: a longitudinal follow-up study	Evaluation	Participants

Comorbidity in family medicine – causal or casual? What is the effect of illness diversity? A longitudinal observational study in primary care	Analysis	None
Longitudinal multimorbidity patterns in elderly population using Hidden Markov Models	Analysis	None
Trends in multimorbidity and polypharmacy in the Flemish-Belgian population between 2000 and 2015	Analysis	None
The TATA survey: The translations of the WAI SR are homogene-ous between Spain, Poland, Slovenia, France and Italy	Development methods	Participants
Does Time Restricted Feeding (16:8) reduce metabolic risk factors in pre-diabetic individuals who suffer from obesity more than the Caloric Restriction diet	Evaluation	Participants
Physical activity prescription	Evaluation	Participants
The immigrant family doctors: The challenges of immigration and the impacts on Primary Care	Analysis	Participants
Evolocumab Versus Ezetimibe in Addition To Statins For Sec-ondary Prevention Of Major Adverse Cardiovascular Events In Patients with Type 2 Diabetes and Hypercholesterolemia	Evaluation	Participants
Relationship between omentin and chemerin levels and meta-bolic indices of obesity within one year in non-morbid over-weight and obese adults	Analysis	Participants
Vulnerable and diabetes patients' perspectives on the advantages of patient education in primary care centre: a qualitative study in France	Needs identification	Participants
Educating Nursing Home Staff to Improve Residents' End-of-life Care and to Reduce Burdensom Hospitalisations - Baseline Findings and Feasibility of a Randomised Controlled Trial	Implementation	Feedback after inter-vention
Effect of time elapsed from the onset of heart failure decom-pensation symptoms to primary care consultation	Analysis	Participants
Reasons of bad adherence to scientific researches among young general practitioners and its improvement	Analyses Research culture in GP/FM	Participants
What influences medical students' choice of family medicine as a career? A research protocol from the 2018/2019 EGPRN Fellows.	Needs identification	Participants

The EGPRN Research Strategy – theory and practice

Esperanza Diaz, Sophie Eliat-Tsanani,
Heidrun Lingner, Radost Assenova

Theoretical Perspective

The research domain of family medicine can be explored from different theoretical perspectives. One of these was presented by Stange in 2001 as a wheel based on the understanding of knowledge in four quadrants crossing each other: the individual, the collective, and the inner and outer reality (Figure 2)[1]. Quadrant 1 represents physician self-knowledge; quadrant 2 represents knowledge of the patient, family, and community; quadrant 3 represents knowing about systems and quadrant 4 represents knowledge of basic physics, chemistry, biology, genetics, and reductionistic understanding of medical processes. At the intersection of the four quadrants is the integrative function of family practice[1].

Green commented on this generalist wheel in 2004 as follows: "Family physicians are initiators of research by constantly identifying challenges and opportunities within their practices. They then seek remedies that are evidence based, just, and placed into context amidst other priorities. Assessing what happens may lead to a revision of ideas and a never-ending quest of improvement. In this framework, the research domain is seen to be derived from practice experience, be about practice, and be used in practice in a recurring cycle. Some would identify in the wheel elements they would label as quality improvement and practice audit, instead of research. Nonetheless, this process reflects a scientific enterprise that can be incorporated into the domain of family medicine research."[2].

Taking the above lines of thought into consideration and based on our review and literature review, we propose to follow a modified research wheel that considers several elements to advance research in GP/FM (Figure 3). The elements of the modified research wheel are:

Figure 2: *Generalist wheel of knowledge, understanding and inquiry[1].*

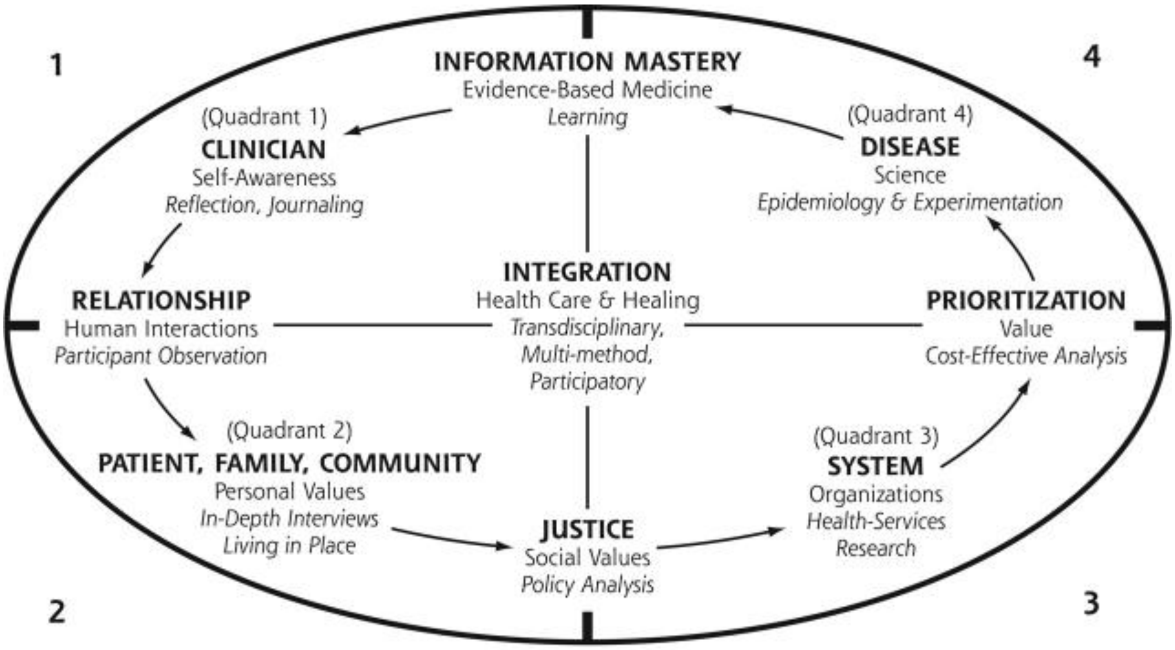

For each item, bold capitalized words on the first line signify **"FOCUS OF KNOWLEDGE,"** normal text on the second line signifies the "Task of Understanding," and italicized words on the third line signify the *"Mode of Inquiry."*

1. Identification of needs
2. Analysis of data
3. Development of new methods
4. Implementation of research: feasibility, cost-effective analyses
5. Evaluation of interventions
6. Scaling-up: improvement of services

Meaningful user involvement and responsible innovation should be considered at all the relevant phases of the development of research. Figure 3 also presents new trends or elements that should be explicitly addressed when considering the strategy for research in GP/FM. Finally, we incorporate the important elements of leadership for capacity building, the development of a research culture in GP/FM, and external funding for research.

Relevant Actions

In the following, we highlight several relevant actions for GP/FM research, related to the circles in the wheel. This list is not intended to be fully exhaustive, and represents only the opinions of the authors following the literature reviews undertaken and consultation with colleagues.

- Identification of needs: what needs to be changed that can be changed?

  – Interdisciplinarity and intersectionality as the "new" trends: The identification of "edges"/interfaces between health professionals and systems that represent a barrier for health care delivery can be the first step for an innovative solution.

  – Alliance with stakeholders from the beginning of the research idea is necessary to choose the adequate outcomes and measures if they are to be implemented.

Figure 3: *Research wheel and proposed elements to consider in order to advance research in GP/FM.*

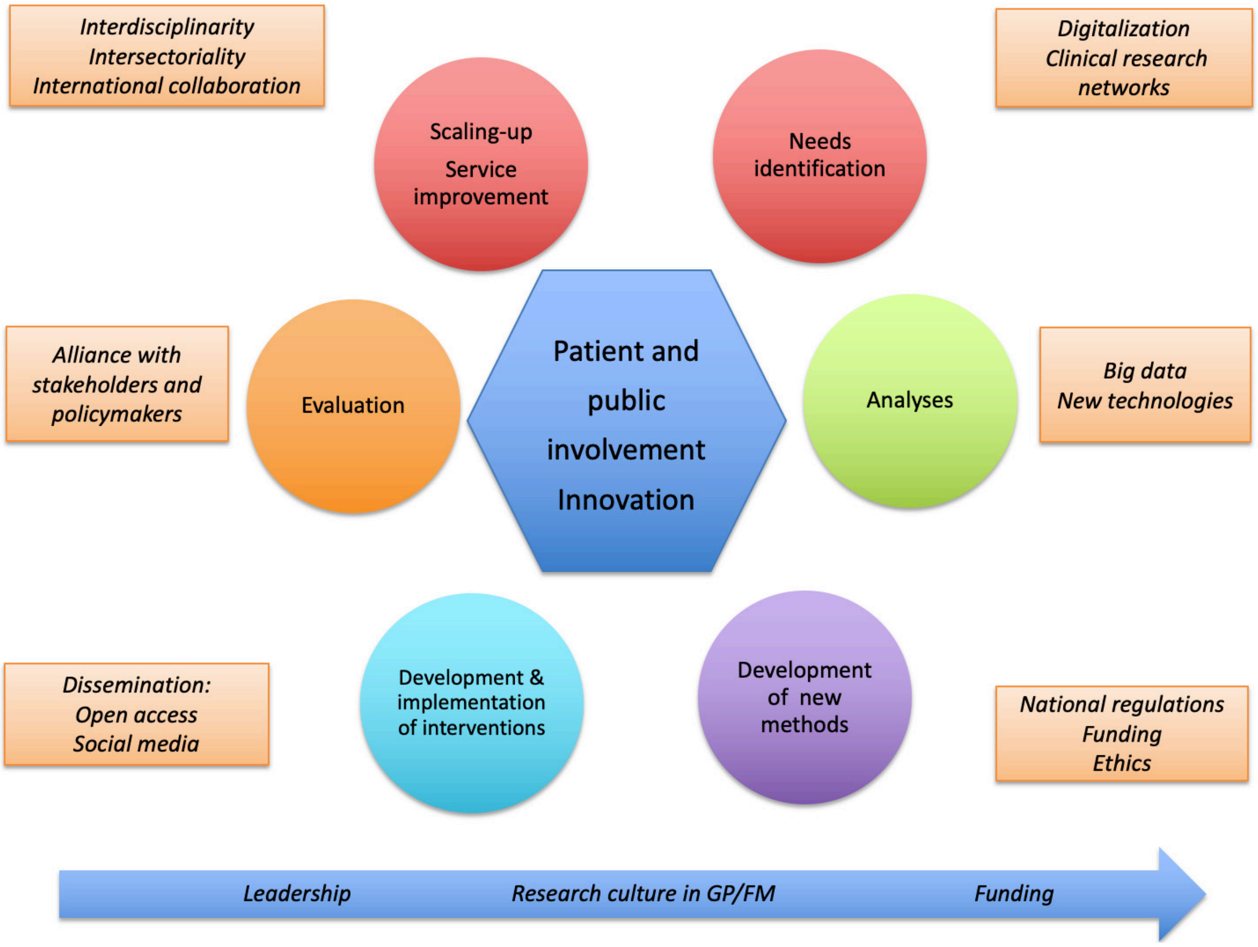

• Analyses and development of new methods: what kind of data do we already possess, what will we "get" in the future, what kind do we need, and what are the new trends in analysing this data?

– Representative samples from a sociodemographic perspective should be the rule in all projects: minorities, frail elderly, vulnerable populations, etc.

– Representative sampling from a disease perspective: multimorbidity versus single/narrow focus (silo thinking)[3].

– Clinical research networks: Use of innovative research methods that optimise the use of already sampled data and possibilities of international comparison and sampling.

– Systematic use of electronic records, automatic extraction of baseline data.

– Harmonising datasets and creation/ definition of a common "core dataset" in order to improve national and international collaboration.

– Interventions using created cohorts of routine (automatically extracted) data should be further developed.

– Collaboration with other health care services: calibration of the GP datasets with the needs of other specialists, home services, etc.

– Big data: collaboration with emerging "Genomics and Metabolomics" fields /personalized medicine, IT and AI specialists, etc. in a way that serves Family Medicine and the general public.

• Development and implementation of interventions: what exactly are we going to test, and is it worth being tested?

– Translational research specifically for primary care as opposed to only for patients attending secondary care is key to developing evidence that is applicable for the GP population.

– Interventions should be built upon both identification of needs and theoretically sound evidence and be systematically described in terms of adaptation and local implementation, to permit the replication of experiments in different settings and further up-scaling.

– Piloting interventions in primary care settings should be the norm before starting RCTs or other studies to change existing treatments.

• Evaluation and scaling-up: how to know what works?

– Complex interventions[4-6] are prevalent in FM/GP. Their evaluation should include the sequential description of the different phases in the development, adaptation and implementation of interventions, as well as feasibility studies[4-6]. The dissemination of this information to the research community and to practitioners and policy makers is indispensable in bridging the gap between knowledge and practice.

– Cost-efficiency studies are needed and should be incorporated in design by default.

– Although there is a growing body of examples of RCTs in primary care, too often the evidence is based on studies conducted with patients in secondary care under specialists, which may or may not be representative of the population in primary care.

– Increasing the participation of GPs and their patients in clinical trials is key.

Overarching themes

• *Meaningful user involvement at the heart of meaningful research*

As mentioned above, user involvement and responsible innovation are considered in our model at all the relevant phases of the development of research. Patient involvement was already on the list of recommendations in the previous agenda.

Indeed, the concept of user is now broader, and depending on the project, users can be GPs, patients, patient representatives, administration, policymakers, NGOs, etc. Users in this sense play an increasingly recognised role in the broader, macro-level context that shapes organizational capacity and willingness to take action to support sustainable implementation of changes in health care settings. In acknowledgement of this, the importance of user involvement in health research has become a compulsory area to include when applying for research funding from several European agencies.

Meaningful, broad user involvement has been described in the literature for some time[1], but is still lacking in many projects. It could be summarised as doing research 'with' or 'by' service users, rather than

'to', 'about' or 'for' service users[7]. Accordingly, Tierney et al. propose to improve and make mainstream meaningful user involvement in primary care research, and suggest explicitly reporting who the users are and the procedures and methodological innovations used to enhance co-governance and dissemination of research processes and findings[8].

The Normalisation Process Theory (NPT) is one of the theoretical backgrounds that are beginning to be used in primary care research in order to improve implementation and sustainability of interventions. This theory covers the whole trajectory from introduction of new practices through to embedding and sustaining them to the point that they are considered routine, or "normalised"[8,9]. The involvement of users seems to be crucial for this purpose, as explained above, and participatory methodologies, designed to support individuals and groups through the development of action plans to shape implementation work, are being proven useful in research in PHC, especially with migrants and other groups difficult to reach in more traditional research[10,11]. The commonality of participatory methodologies is that they include stakeholders affected by the issue under consideration and in a position to act on the findings[12]. Participatory health research and specific approaches such as Participatory Learning and Action research (PLA)[13] can enhance patient engagement and support implementation processes in PHC.

- *Responsible innovation*

Innovation can raise questions and dilemmas and is often ambiguous in terms of purposes and motivations and unpredictable in terms of impacts[14]. A responsible innovation approach is thus needed to create and report research processes to explore these aspects of innovation in an open, inclusive and timely way. Our considerations regarding this theme are:

- Innovation is not only about technology. Innovative projects in GP/FM include for example:

- new ways of reaching representative populations, including vulnerable populations
- new methods to gather and analyse existing data through clinical research networks
- new interventions to improve trajectories for patients
- development of better collaboration in primary care across disciplines and sectors
- development of new methods of enhancing media "awareness" and improving its use for GPs' purposes, such as patient education, continuous communication between professionals and doctor-patient exchange.

  - Data collection and projects should be ethical and adhere to data protection requirements: The General Data Protection Regulation (GDPR)[15] should be acknowledged and respected as increasing automatic monitoring of patients through smart phones and similar technology also increases the possibility of using data for aims that do not serve all patients or even using data against vulnerable patients.
  - At the same time it is necessary to find ways to facilitate international research collaborations and data exchange in GP/FM.
  - Research in GP/FM should be gentle on resources (human and environmental).

- *Leadership, Research culture in GP/FM and Funding*

  - Good leadership is necessary to create, maintain and improve the visibility and quality of research in GP/FM within medicine in Europe.
  - Capacity building: As a community of researchers, we should collaborate to create academic departments of GP/FM in all medical universities in Europe.
  - Research culture in GP/FM: It is necessary to have researchers in GP, but we also need to improve GPs' basic research understanding in order to enable them to identify needs, appraise published research and estimate its usability and impact on their daily practice-work and therapeutic-related outcomes. We need to build capacity among non-researcher GPs in this respect and not treat them solely as data collection sites.
  - International collaboration in capacity building: countries with more resources should actively invite other countries to collaborate on research projects in order to build capacity. Collaboration also increases the possibilities of getting funding and increases the impact of published papers.

– Coordination and synergies are required to increase opportunities for collaboration and reduce how often the same research questions and challenges within GP/FM are considered in several European countries at the same time, with no or too little knowledge of each other's projects, missing opportunities for collaboration and synergy.

– As a research community, working for as many open access papers and journals as possible should be a priority. Related to this is the issue of the increasing difficulty of getting peer reviewers for publications, which, if not addressed, might in the long run decrease the number of open access publications or increase the cost for the individual researcher.

Conclusion

Considering the wheel of research that we propose in this model, more effort should be put into the left part of the wheel: from reporting the development and implementation of interventions to their evaluation and scaling-up.

Meaningful user involvement, that is to say, including users and relevant stakeholders in the whole research wheel beginning with the definition of the research question all the way to the assessment of the suitability of the methods to be used, is yet to become the rule rather than the exception in primary care research. Alliance with stakeholders is also necessary at several stages of the research wheel in order to achieve responsible innovation, choose the outcomes and measures that can be realistically implemented, improve dissemination of the findings and scale-up evidence-based solutions.

The relevant actions required in each country should be identified and prioritised. We have mapped these actions to the four goals of this strategy thereby permitting the EGPRN to identify how it can support its members. Working to improve leadership, to build capacity, and to increase international collaboration and networking are fundamental in this regard.

References

1. Stange KC, Miller WL, McWhinney I. Developing the knowledge base of family practice. Fam Med. 2001;33(4):286-297.
2. Green LA. The research domain of family medicine. Ann Fam Med. 2004;2 Suppl 2:S23-29.
3. Abbasi K. Generalism for specialists: a medical reformation. BMJ. 2020:368.
4. Moore GF, Audrey S, Barker M, Bond L, Bonell C, Hardeman W, et al. Process evaluation of complex interventions: Medical Research Council guidance. BMJ. 2015;350:h1258.
5. Craig P, Dieppe P, Macintyre S, Michie S, Nazareth I, Petticrew M. Developing and evaluating complex interventions: the new Medical Research Council guidance. Int J Nurs Stud. 2013;50(5):587-92.
6. Fletcher A, Jamal F, Moore G, Evans RE, Murphy S, Bonell C. Realist complex intervention science: Applying realist principles across all phases of the Medical Research Council framework for developing and evaluating complex interventions. Evaluation. 2016;22(3):286-303.
7. National Institute for Health Research (NIHS). NIHR Involve. www.invo.org.uk
8. Tierney E, McEvoy R, O'Reilly-de Brun M, de Brun T, Okonkwo E, Rooney M, et al. A critical analysis of the implementation of service user involvement in primary care research and health service development using normalization process theory. Health expectations: an international journal of public participation in health care and health policy. 2016;19(3):501-15.
9. May CR, Cummings A, Girling M, Bracher M, Mair FS, May CM, et al. Using Normalization Process Theory in feasibility studies and process evaluations of complex healthcare interventions: a systematic review. Implementation science : IS. 2018;13(1):80.
10. O'Reilly-de Brun M, MacFarlane A, de Brun T, Okonkwo E, Bonsenge Bokanga JS, Manuela De Almeida Silva M, et al. Involving migrants in the development of guidelines for communication in cross-cultural general practice consultations: a participatory learning and action research project. BMJ Open. 2015;5(9):e007092.
11. Teunissen E, Gravenhorst K, Dowrick C, Van Weel-Baumgarten E, Van den Driessen Mareeuw F, de Brun T, et al. Implementing guidelines and training initiatives to improve cross-cultural communication in primary care consultations: a qualitative participatory European study. Int J Equity Health. 2017;16(1):32.
12. www.ICPHR.org
13. de Brun T, O'Reilly-de Brun M, Van Weel-Baumgarten E, Burns N, Dowrick C, Lionis C, et al. Using Participatory Learning & Action (PLA) research techniques for inter-stakeholder dialogue in primary healthcare: an analysis of stakeholders' experiences. Res Involv Engagem. 2017;3:28.
14. Council EaPSR. Framework for Responsible Innovation 2017 [Available from: https://www.epsrc.ac.uk/research/framework/.
15. General Data Protection Regulation (EU) 2016/679. https://gdpr.eu

The Way Forward in Summary

Claire Collins

A strong research basis is a necessity[1,2] and it is accepted that research in general practice is important in terms of improving patient outcomes[3].

If we are to continue to improve the quantity, quality, impact and co-ordination of GP/FM research, a key concept noted here and in the previous EGPRN Research Agenda[4] is that of capacity. Research capacity building (RCB) is critical at the individual, organisational and environmental level[5]. Cooke's framework is useful to measure progress and evaluate the impact of RCB highlighting six principles cutting across the activities undertaken at different structural levels[6]. EGPRN and WONCA members' views on the strategies for RCB have been documented elsewhere[7].

It is necessary to consider what the knowledge deficits are and to set research priorities[8,9]. Ideally these should be agreed and co-ordinated at a national level. Prioritisation is needed to ensure the efficient use of limited resources in order to meet national and international objectives while showing benefit to society. There are a number of approaches to prioritisation, however, priorities change over time[8,10] and the process should allow for this and should include methodological priorities along with topics[10]. Concepts such as inclusiveness, evaluation and transparency are critical[11].

Knowledge transfer and exchange (KTE) is an important component ensuring a process of exchange between researchers and knowledge users. The Evidence-based model for the Transfer and Exchange of Research Knowledge (EMTReK) is a possible functional model of KTE to ensure knowledge transfer activities are incorporated from the earliest phase of the research process, are appropriate and an evaluation framework is included[12]. Meaningful PPI is essential[13].

GP/FM researchers need to establish and engage in national and international networks[7, 14, 15] and in particular those that bring a range of disciplines together to ensure the successful delivery of a portfolio of high-quality studies. It is through such endeavours that we can drive new knowledge, play an integral role in translational research and fully participate in the laboratory-community-laboratory pathway[16]. Innovation is required[17] but adaption to different settings and health systems must take account of the specific context[17,18].

Meaningful user involvement should be considered at all phases of the development of research. However, it may be difficult to find suitable patients or patient representatives for individual projects. Making use of existing toolkits[19,20] and engaging with national and international patient platforms[21] and representative groups is necessary.

We need to continue to conduct primary care clinical studies dealing with common, everyday complaints and illnesses as recommended in the 2009 Research Agenda[4]. Collaborative funding proposals on a European level should ideally be sought for such projects.

The relevant actions required in each country should be identified and prioritised. We have mapped these actions to the four goals of this strategy thereby permitting the EGPRN to identify how it can support its members. Working to improve leadership, to build capacity, to support the creation of a research culture in GP/FM and to increase international collaboration and networking are fundamental in this regard.

References

1. Beasley JW, Starfield B, van Weel C, Rosser WW, Haq CL. Global health and primary care research. J Am Board Fam Med. 2007; 20:518–526.
2. van Weel C, Rosser WW. Improving health care globally: a critical review of the necessity of family medicine research and recommendations to build research capacity. Ann Fam Med. 2004;2:S5–S16.
3. Mant D, Del Mar CB, Glasziou P, Knotternerus A, Wallace P, van Weel C. The state of primary care research. Lancet 2004; 364:1004-1006.
4. Hummers-Pradier E, Beyer M, Chevallier P, et al. The Research Agenda for general practice/family medicine and primary health care in Europe. European General Practice Network (EGPRN). Maastricht, 2009.
5. Cooke J, Ariss S, Smith C, Read J. On-going collaborative priority-setting for research activity: a method of capacity building to reduce the research-practice translational gap. Health Res Policy Syst 2015;13:25.

6. Cooke JA. A framework to evaluate research capacity building in health care. BMC Fam Pract 2005; 6(1):6-44.

7. Huas C, Petek D, Diaz E, Muñoz-Perez MA, Torzsa P, Collins C. Strategies to improve research capacity across European general practice: The views of members of EGPRN and Wonca Europe. Eur J Gen Pract. 2019 Jan;25(1):25-31. doi: 10.1080/13814788.2018.1546282.

8. Burgers JS, Wittenberg J, Keuken DG, et al. Development of a Research Agenda for general practice based on knowledge gaps identified in Dutch guidelines and input from 48 stakeholders. Eur J Gen Pract. 2019;25:19–24.

9. Research and Development Directorate, Department of Health (UK). Best Research for Best Health A new national health research strategy. London: Department of Health. Jan 2006.

10. Vinker S, Ungan M. Research Agenda in family medicine – should we adopt the Dutch approach? Eur J Gen Pract. 2019;25(1):5–6.

11. Viergever RF, Olifson S, Ghaffar, Terry RF. A checklist for health research priority setting: nine common these of good practice. Health Research Policy and Systems 2010; 8:36. http://www.health-policy-systems.com/content/8/1/36.

12. Prihodova L, Guerin S, Tunney C, Kernohan WG. Key components of knowledge transfer and exchange in health services research: Findings from a systematic scoping review. J Adv Nurs 2019; 72(2): 313-326.

13. National Institute for Health Research (NIHS). NIHR Involve. www.invo.org.uk

14. Sullivan F, Butler C, Cupples M, Kinmonth AL. Primary care research networks in the United Kingdom. BMJ 2007; 334: 1093.

15. van der Zee J, Kroneman M, Bolíbar B. Conditions for research in general practice. Can the Dutch and British experiences be applied to other countries, for example Spain? Eur J Gen Pract 2003; 9:41–47.

16. Mainous AG, Baker R. Primary care research: time for some new questions? Quality in Primary Care 2009; 17:161-163.

17. TO-REACH. Transferring Innovation in Health Systems. Advanced version of the Strategic Research Agenda. May 2019. Ref. Ares(2019)7788239 - 18/12/2019.

18. Greenhalgh T, Robert G, Macfarlane F, Bate P, Kyriakidou O. Diffusion of innovations in service organizations: systematic review and recommendations. Milbank Q. 2004;82:581-629.

19. Bagley HJ, Short H, Harman NL et al. A patient and public involvement (PPI) toolkit for meaningful and flexible involvement in clinical trials – a work in progress. *Res Involv Engagem* 2, 15 (2016). https://doi.org/10.1186/s40900-016-0029-8.

20. About - EUPATI - Dedicated Training Provider.

21. National Platforms - EUPATI.

Acknowledgements

We would like to acknowledge the assistance of the librarians in the Irish College of General Practitioners (ICGP) – Gillian Doran and Patricia Patton – for their assistance with the literature searches. We extend our thanks to Colleen O'Neil in the ICGP also for cross-referencing and proof-reading the document. We are grateful to the members of the EGPRN Council who provided feedback on the previous versions of this document. We appreciate the support and guidance of the EGPRN Executive Board members.

EGPRN
